# A two-stage microbial association mapping framework with advanced FDR control

**DOI:** 10.1186/s40168-018-0517-1

**Published:** 2018-07-25

**Authors:** Jiyuan Hu, Hyunwook Koh, Linchen He, Menghan Liu, Martin J. Blaser, Huilin Li

**Affiliations:** 1 Division of Biostatistics, Department of Population Health, New York University School of Medicine, New York, NY 10016 USA; 2 Shanghai Center for Mathematical Sciences, Fudan University, Shanghai, 200433 China; 3 Department of Medicine, New York University School of Medicine, New York, NY 10016 USA

**Keywords:** Microbiome, Two-stage microbial association mapping, Taxonomic tree, Microbial group association test, False discovery rate, Hierarchical BH, Selected subset testing

## Abstract

**Background:**

In microbiome studies, it is important to detect taxa which are associated with pathological outcomes at the lowest definable taxonomic rank, such as genus or species. Traditionally, taxa at the target rank are tested for individual association, followed by the Benjamini-Hochberg (BH) procedure to control for false discovery rate (FDR). However, this approach neglects the dependence structure among taxa and may lead to conservative results. The taxonomic tree of microbiome data represents alignment from phylum to species rank and characterizes evolutionary relationships across microbial taxa. Taxa that are closer on the tree usually have similar responses to the exposure (environment). The statistical power in microbial association tests can be enhanced by efficiently employing the prior evolutionary information via the taxonomic tree.

**Methods:**

We propose a two-stage microbial association mapping framework (massMap) which uses grouping information from the taxonomic tree to strengthen statistical power in association tests at the target rank. massMap first screens the association of taxonomic groups at a pre-selected higher taxonomic rank using a powerful microbial group test OMiAT. The method then proceeds to test the association for each candidate taxon at the target rank within the significant taxonomic groups identified in the first stage. Hierarchical BH (HBH) and selected subset testing (SST) procedures are evaluated to control the FDR for the two-stage structured tests.

**Results:**

Our simulations show that massMap incorporating OMiAT and the advanced FDR controlling methodologies largely alleviates the multiplicity issue. It is statistically more powerful than the traditional association mapping directly at the target rank while controlling the FDR at desired levels under most scenarios. In our real data analyses, massMap detects more or the same amount of associated species with smaller adjusted *p* values compared to the traditional method, which further illustrates the efficiency of the proposed framework. The R package of massMap is publicly available at https://sites.google.com/site/huilinli09/software and https://github.com/JiyuanHu/.

**Conclusions:**

massMap is a novel microbial association mapping framework and achieves additional efficiency by utilizing the intrinsic taxonomic structure of microbiome data.

**Electronic supplementary material:**

The online version of this article (10.1186/s40168-018-0517-1) contains supplementary material, which is available to authorized users.

## Background

The microbiome has important interactions in human health and disease [1]. Microbiota disturbance has been associated with human diseases including obesity, diabetes, Crohn’s disease, and numerous other conditions [2–5]. With the development of next-generation sequencing techniques, it is feasible to extract all the microbiota from multiple parts of the human body, assess microbiome composition, and then link it with human health/diseases [6]. Two high-throughput parallel sequencing approaches are widely used; one targeted to the 16S rRNA amplicon sequencing and the other to metagenomic shotgun sequencing [7].

We introduce our method using microbiome data generated from 16S rRNA sequencing studies. Based on 16S rRNA sequencing, the reads from the amplicons are clustered into operational taxonomic units (OTUs) according to sequence similarity, and then their read counts or relative abundances are evaluated. OTUs are assigned to a taxonomic tree at the kingdom, phylum, class, order, family, genus, and species ranks [8, 9], hierarchically using either the online Greengenes [10] or the RDP classifier [11] taxonomy assignments. The taxonomic tree displays the evolutionary relationship among the microbial taxa; taxa that are closer on the tree tend to have similar responses to environmental shifts [12, 13].

There is general interest to detect association between traits and microbial taxa at the various taxonomic ranks. Researchers usually begin investigation for community-level analysis at the highest taxonomic rank to determine whether the overall microbial profiles are different between groups or associated or not with the trait. For example, the analyses of bacterial communities within (α-diversity) and between samples (β-diversity) [2, 3, 14] are two commonly used approaches. Significant results at the community level lead to the further identification of the roles of specific microbes to better understand the mechanisms involved in microbiome perturbations. Most often, investigators are interested in the association mapping of taxa at the lowest definable rank, such as genus or species, considered as the ‘target rank.’

Several statistical methods have been developed explicitly for microbiome data to examine for differential abundance among groups or to test for microbial association with continuous traits at a specific taxonomic rank [15–18]. LEfSe [15] uses the Kruskal-Wallis test to detect significant differential abundances among groups, but does not correct for multiple comparisons. Other association testing methods, such as metagenomeSeq-fit Zig [16] and STAMP [17], assume that the testing taxa are independent. They examine taxa individually and use the *q* value method [19] or the Benjamini-Hochberg (BH) procedure [20] to control the false discovery rate (FDR). Because of the sparse signal and large number of multiple comparisons, this usually leads to very few discoveries. However, the microbes in a community are usually dependent upon one another, and trait-associated taxa tend to be clustered evolutionarily instead of randomly distributed across the community [12]. Therefore, an association mapping framework which could exploit the known taxonomic structure, i.e., the microbial evolutionary relationships, to better target on associated taxa would have substantial potential.

In this study, we propose a powerful two-stage microbial association mapping framework (massMap), which incorporates advanced FDR-controlling procedures based on the microbial dependence structures through the taxonomic tree. In the first stage, an upper-level taxonomic rank is first pre-selected as the ‘screening rank.’ The association for taxonomic groups at this selected rank is then tested by OMiAT, a new microbiome-based group association testing method, designed to discover significant association signals for an upper-level taxon considering various relative contributions from both microbial abundance and phylogenetic information in its lower lineages [18]. In the second stage, the association tests for taxa within the groups discovered in the first stage are performed at the ‘target rank.’

The proposed framework constitutes three building components: (1) a pre-selected taxonomic rank for screening; (2) a powerful microbial group test OMiAT, to identify the taxonomic groups that contain the associated taxa; and (3) an advanced FDR-controlling methodology to resolve the dependency among taxa. The taxonomic tree classifies the microbes in the bacterial kingdom into ranks from the most general rank phylum where each taxonomic group contains many taxa, to the most specific rank species with one member in each group. Selection of a screening rank balances the group testing power as well as proportion of truly associated taxa among the significant groups. The group association test for screening is more powerful at a higher rank regarding. However, as the groups classified by the higher rank have too many members, there is still a very high proportion of unassociated taxa within the groups, i.e., the signal has not condensed enough after screening. Thus, a middle taxonomic rank such as order or family is expected to perform best in the proposed two-stage framework. A highly powerful microbial group test guarantees a higher probability that it only eliminates not-associated microbial taxa and retains true signals to the target rank. The data-driven approach of OMiAT is a microbial group test designed for this purpose and it can efficiently detect microbial groups varying in association patterns. The hierarchical BH (HBH) [21, 22] and the selected subset testing (SST) procedures [23, 24] are two FDR-controlling procedures capable of handling multiple hypotheses with two-stage structures. These have been applied in microarray data analysis and permit greater discovery than the traditional BH procedure [25].

massMap fully utilizes the prior information from the taxonomic tree, in which the first stage eliminates less-promising taxa and therefore condenses the association signal. Through extensive simulations and two real data analyses, we show that massMap achieves higher statistical power and detects more biologically meaningful taxa than the traditional one-stage microbial association test.

## Methods

### A two-stage microbial association testing framework: massMap

Suppose we have observed the microbial abundance information of *N* subjects for *M* taxa at the target rank. We propose to perform the screening test at a pre-selected higher screening rank, and its taxonomy assignment partitions *M* taxa into *G* taxonomic groups. Let the *g*th group consist of *m*_*g*_ (*m*_*g*_ ≥ 1) taxa then ∑_*g*_*m*_*g*_ = *M*. For subject *i*,  denote the outcome trait, either binary or continuous, as *Y*_*i*_, the abundance of taxa in the *g*th group as ${\boldsymbol{Z}}_{ig}={\left({Z}_{ig1},{Z}_{ig2},\dots, {Z}_{ig{m}_g}\right)}^{\prime },$ and *p* covariates as ***X***_*i*_ = (*X*_*i*1_, *X*_*i*2_, …, *X*_*ip*_)^′^ respectively.

At the screening rank, the group association test is used to examine the association between each group of taxa and the outcome trait. We use the logistic regression model for a binary outcome:

1 $$ \mathrm{Logit}\left[\mathrm{P}\left({Y}_i=1\right)\right]={\beta}_0+{\boldsymbol{\alpha}}^{\prime }{\boldsymbol{X}}_i+{\boldsymbol{\beta}}_g^{\prime }{\boldsymbol{Z}}_{ig}, $$

and the linear regression model for a continuous outcome:

2 $$ {Y}_i={\beta}_0+{\boldsymbol{\alpha}}^{\prime }{\boldsymbol{X}}_i+{\boldsymbol{\beta}}_g^{\prime }{\boldsymbol{Z}}_{ig}+{\epsilon}_i. $$

where *β*_0_ is the intercept, ***α*** = (*α*_1_, …, *α*_*p*_)^′^ is the vector of coefficients of covariates, ${\boldsymbol{\beta}}_g={\left({\beta}_{g1},{\beta}_{g2},\dots, {\beta}_{g{m}_g}\right)}^{\prime }$ is the vector of coefficients for abundance of taxa from group *g*, and *ϵ*_*i*_ is an error term with mean 0 and variance *σ*^2^. The definition of a trait-associated group is that at least one of the taxa in the group is associated with the trait. Correspondingly, the screening hypothesis for the *g*th group is

$$ {H}_{0g}:{\beta}_{g1}={\beta}_{g2}=\dots ={\beta}_{g{m}_g}=0\kern0.5em v.s.{H}_{1g}:\mathrm{at}\ \mathrm{least}\ \mathrm{one}\ {\beta}_{gj}\ne 0,\kern1em j=1,\dots, {m}_g. $$

OMiAT is a powerful test specifically designed for the detection of varying association patterns for a group of taxa and can accommodate multiple covariates [18]. Thus, it is a useful screening test for our two-stage association mapping framework, and we employ it to test the associations between taxonomic groups and traits. The corresponding test statistic is

$$ {M}_{OMiAT}^g=\mathrm{minP}\left\{{T}_{aSPU}^g,{Q}_{OMiRKAT}^g\right\}. $$

where ${T}_{a\mathrm{SPU}}^g$ and ${Q}_{OMiRKAT}^g$ are two adaptive test statistics. ${T}_{a\mathrm{SPU}}^g$ is useful for modulating different association patterns arising from highly imbalanced microbial abundances. It is adapted from the sum of score powered tests (SPU) [26] which was originally proposed for gene- or region-based association testing in genome-wide association studies [18]. ${Q}_{OMiRKAT}^g$, advantageous in detecting microbial group associations utilizing phylogenetic tree information, is tailored from the microbiome regression-based kernel association test (MiRKAT) [27], originally proposed as a microbial community association test. Please see Eq. (9) of [18] for notation and detailed explanation. OMiAT aims to detect varying association patterns which can be captured by either aSPU or OMiRKAT, using the minP procedure within the taxonomy group.

At the target rank, we are interested in the association between each taxon and the outcome trait. For taxon *j*(*j* = 1, …, *m*_*g*_) from the *g*th group, the model for binary, and continuous trait are

$$ \mathrm{Logit}\left[\mathrm{P}\left({Y}_i=1\right)\right]={\beta}_0+{\boldsymbol{\alpha}}^{\prime }{\boldsymbol{X}}_i+{\beta}_{gj}{Z}_{igj}, $$

and

$$ {Y}_i={\beta}_0+{\boldsymbol{\alpha}}^{\hbox{'}}{\boldsymbol{X}}_i+{\beta}_{gj}{Z}_{igj}+{\epsilon}_i. $$

respectively. Thus, the corresponding targeting hypotheses within group *g* are

$$ {H}_{0g1}:{\beta}_{g1}=0\kern0.5em \mathrm{vs}\kern0.5em {H}_{1g1}:{\beta}_{g1}\ne 0, $$

…

$$ {H}_{0 gj}:{\beta}_{gj}=0\kern0.5em \mathrm{vs}\ {H}_{1 gj}:{\beta}_{gj}\ne 0, $$

…

$$ {H}_{0g{m}_g}:{\beta}_{g{m}_g}=0\kern0.5em \mathrm{vs}\ {H}_{1g{m}_g}:{\beta}_{g{m}_g}\ne 0. $$

Denote the predicted value of *Y*_*i*_ under *H*_0*gj*_ by ${\widehat{Y}}_i$, where ${\widehat{Y}}_i={\widehat{\beta}}_0+{\widehat{\boldsymbol{\alpha}}}^{\prime }{\boldsymbol{X}}_i$ for continuous traits and ${\widehat{Y}}_i=\mathrm{Logi}{\mathrm{t}}^{-1}\left({\widehat{\beta}}_0+{\widehat{\boldsymbol{\alpha}}}^{\prime }{\boldsymbol{X}}_i\right)$ for binary traits, respectively; ${\widehat{\beta}}_0$ and $\widehat{\boldsymbol{\alpha}}$are the maximum likelihood estimates (MLEs) under *H*_0*gj*_. The association between the *j*th taxon and the trait can be tested by the non-parametric score test statistic [18]:

3 $$ {\mathrm{U}}_{gj}=\sum \limits_{i=1}^N\left({Y}_i-{\widehat{Y}}_i\right){Z}_{igj},j=1,\dots, {m}_{g.} $$

We used the residual-permutation method to calculate *p* values of statistics as in [18].

**Fig. 1:**
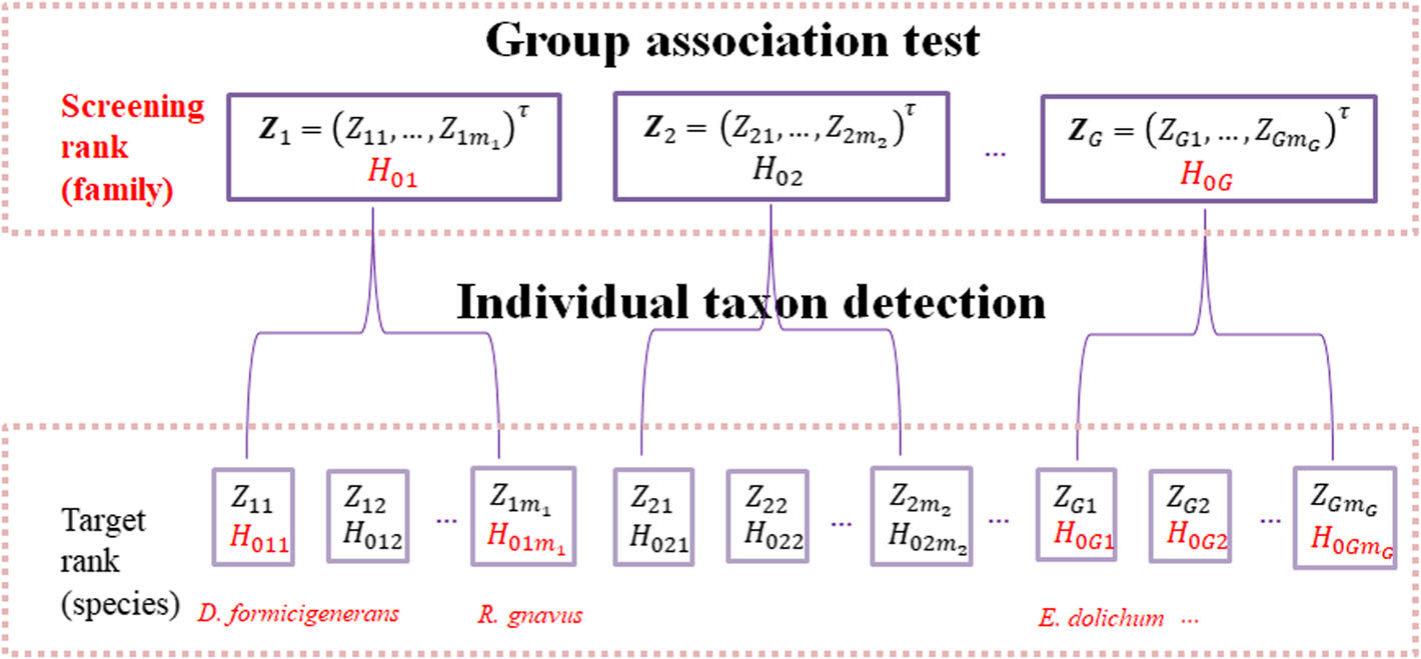
Schematic of the two-stage microbial association mapping framework massMap. The association testing hypotheses are organized into a two-level tree. The screening test is conducted at the first level of the tree using OMiAT (proposed) or the aggregated method (competing method). Two advanced FDR controlling procedures are evaluated in this framework: the hierarchical BH (HBH) and the selected subset testing with BH (SST) procedure [23, 24]

The two-stage microbial association mapping framework massMap is described as a two-level hierarchical tree in Fig. 1. The group association test and the individual taxon detection are performed at the screening rank and the target rank respectively. massMap screens out groups among which taxa are unlikely to be associated and only retains the more promising ones to the target rank. The association signal condenses after the screening step so that the proportion of truly associated taxa is increased within the candidate groups. By utilizing the advanced FDR controlling procedures introduced below, we could further enhance the association mapping power. Next, we discuss how to implement the advanced FDR controlling procedures to move from the screening rank to the target rank to discover associated taxa.

### Advanced FDR controlling procedures

We consider two advanced FDR-controlling procedures: the hierarchical BH (HBH) procedure [21, 22] and the selected subset testing with BH (SST) procedure [23, 24] to accommodate the hierarchically structured hypotheses in massMap. Both procedures involve the assembly of multiple BH procedures. All of the BH procedures below are conducted at level FDR = *q* without explicit declaration. Denote the set of screening hypotheses by ${\mathcal{T}}_0=\left\{{H}_{01},\dots, {H}_{0g},\dots, {H}_{0G}\right\}$ and the target hypotheses organized into groups by ${\mathcal{T}}_1=\left\{{H}_{011},\dots, {H}_{01j},\dots, {H}_{01{m}_1}\right\},\dots,$ ${\mathcal{T}}_G=\left\{{H}_{0G1},\dots, {H}_{0 Gj},\dots, {H}_{0G{m}_G}\right\}$. Further, the corresponding raw *p* values are denoted by ${\mathcal{P}}_0=\left\{{p}_{01},\dots, {p}_{0g},\dots, {p}_{0G}\right\}$, ${\mathcal{P}}_1=\left\{{p}_{011},\dots, {p}_{01j},\dots, {p}_{01{m}_1}\right\},\dots,$ and ${\mathcal{P}}_G=\left\{{p}_{0G1},\dots, {p}_{0 Gj},\dots, {p}_{0G{m}_G}\right\}$, respectively.

#### HBH procedure

The HBH procedure applies to *p* values arranged in a tree of disjoint subfamilies and is conducted as follows:

- ▪ At the screening rank: apply the BH procedure to ${\mathcal{P}}_0$. The groups with adjusted *p* values <*q* are called the discovered groups. Without loss of generality, we assume that the first *R* groups are discovered;
- ▪ At the target rank: within the *g*th discovered group, *g* = 1, ⋯, *R*, apply the BH procedure to ${\mathcal{P}}_g$. The taxa with adjusted *p* values <*q* are reported as trait-associated taxa.

#### SST procedure

The SST procedure is to treat the two-level hierarchical tree simply as a two-stage structure, and it can be implemented as follows:

- ▪ At the screening rank: same as in the HBH procedure;
- ▪ At the target rank: pool the *p* values from *R* discovered groups into one set and denote it as ${\mathcal{P}}_{\mathrm{pooled}}=\left\{{p}_{011},\dots, {p}_{01{m}_1},\dots, {p}_{0R1},\dots, {p}_{0R{m}_R}\right\}$. The corresponding taxa are called the selected subset. Apply the BH procedure to the pooled *p* values. The taxa with adjusted *p* values <*q* are reported as trait-associated taxa.

The HBH procedure has merit since it reports many discoveries [21, 22], but it sometimes has higher FDR than the nominal level. In comparison, SST is more conservative than HBH. If tests between two stages are independent, either utilizing an independent source of data or testing unrelated hypotheses, SST procedure can control the FDR at the desired level. Notice that in our setting, we use the same data to implement the tests in both stages; the test statistics in the second stage might thus not be independent of those in the first stage. As the SST procedure is commonly implemented in microarray analysis where tests from both stages use the same data [25], we ignore the minor dependence among tests from both stages and apply the SST procedure to the microbial association mapping analysis. For convenience, we denote the two-stage framework with OMiAT as the screening test and HBH or SST as the FDR-control procedures as OMiAT-HBH or OMiAT-SST, respectively.

### Aggregated methods

Apart from OMiAT [18], there are other methods to detect microbial group associations which are termed aggregated methods in this article, including commonly used programs such as LEfSe [15], metagenomeSeq-fit Zig [16], and STAMP [17]. Even though the modeling techniques and test statistics used are different, they all assume that the effect sizes and directions of all taxa within the tested group are the same. Under this assumption, the abundance within each tested group is summed and regressed towards the outcome. Here, we investigate a representative aggregated method, as illustrated in [18]. Specifically, for the *g*th group, the corresponding logistic regression model for binary outcome is

$$ \mathrm{Logit}\left[\mathrm{P}\left({Y}_i=1\right)\right]={\beta}_0+{\boldsymbol{\alpha}}^{\prime }{\boldsymbol{X}}_i+{\beta}_g^{\ast}\sum \limits_j{Z}_{igj}, $$

and the linear regression model for the continuous outcome is

$$ {Y}_i={\beta}_0+{\boldsymbol{\alpha}}^{\prime }{\boldsymbol{X}}_i+{\beta}_g^{\ast}\sum \limits_j{Z}_{igj}+{\epsilon}_i $$

where ${\beta}_g^{\ast }$ is the shared coefficient for the taxa in group *g*, which reflects the assumption of the aggregated methods: the effect sizes and directions of all taxa within the tested group are the same. Then, its screening hypothesis is

$$ {H}_{0g}:{\beta}_g^{\ast }=0\ \mathrm{vs}\kern0.5em {H}_{1g}:{\beta}_g^{\ast}\ne 0. $$

The non-parametric score test statistic from Eq. (3) is used to test this screening hypothesis. As a comparison, we include this test in the simulations and real data analyses and refer to it as the aggregated method. We denote the two-stage framework with the aggregated method as the screening test and HBH or SST as FDR-control procedures as AGG-HBH or AGG-SST, respectively.

### Traditional one-stage method

The traditional microbial association tests do not require a screening step, rather directly conducting the association test between the trait and taxa one by one at the target rank. Therefore, the hypotheses set is the union of hypotheses from *G* groups, i.e., $\mathcal{T}={\mathcal{T}}_1\cup \dots \cup {\mathcal{T}}_G$. The corresponding *p* value set are denoted by $\mathcal{P}={\mathcal{P}}_1\cup \dots \cup {\mathcal{P}}_G$, and are calculated from the non-parametric score statistics in Eq. (3). In traditional methods, the BH procedure is most commonly used to control FDR for multiple comparisons. We used the traditional method as the benchmark method in both the simulations and real data analyses, and denoted it as BH.

## Results

We first conducted comprehensive simulations to evaluate the performance of OMiAT-HBH, OMiAT-SST, AGG-HBH, AGG-SST, and BH methods in relation to their false discovery rates (FDR) and true positive rates (TPR) for identifying the associated taxa at the target rank. Then, we further applied those methods to two real microbiome studies to compare their practical performance: one involving the American Gut Project (AGP) (www.americangut.org) and the other, a two-group murine study [28].

### Simulation settings

The simulation settings were similar to those used in prior studies [18, 27, 29]. In those studies, the abundance tables were first generated from the Dirichlet-multinomial (DM) distribution based on a real microbiome dataset. Then, the generalized linear model was utilized to generate the value of outcome traits. OTUs were partitioned into clusters using the partitioning-around-medoids (PAM) clustering algorithm [30], where OTUs from a certain number of clusters were further assigned to be trait-associated. Following those prior studies, we generated our simulation data as below. The taxonomic and phylogenetic tree information and OTU table from AGP’s baseline microbiome data are the basis of our simulated data. In our AGP data analysis, there are 174 OTUs retained after the filtering. We generated the OTU abundance from the DM distribution with 15,000 total reads per sample, using function dirmult() from the R package “dirmult” [31]. The corresponding proportion means and dispersion parameters of the DM distribution were estimated from AGP’s baseline microbiome data.

The continuous and binary traits were generated under the following linear model (4) and logistic regression model (5), respectively,

4 $$ {Y}_i={\sum}_{j\in \Lambda}{\beta}_j\mathrm{scale}\left({\mathrm{Z}}_{ij}\right)+{\upepsilon}_i $$

5 $$ \mathrm{Logit}\ \left[\mathrm{P}\left({Y}_i=1|{\boldsymbol{Z}}_i\right)\right]=\sum \limits_{j\in \varLambda }{\beta}_j\mathrm{scale}\left({Z}_{ij}\right) $$

where *ϵ*_*i*_ ∼ *N*(0, 1) is the error term, and *Z*_ij_ is the OTU abundance for subject *i* = 1, …, *N*. ***β =*** (*β*_1_, …, *β*_*j*_, …, *β*_|*Λ*|_)^′^ is a vector of coefficients for the associated OTU. *Λ* is a set of the indices of truly associated OTU and ∣*Λ*∣ is the number of associated OTU.

To estimate the empirical false discovery rate (FDR) and true positive rate (TPR) at the target rank, i.e., the OTU level, we assigned 17 OTUs (10%) as the truly associated taxa as follows. We first partitioned the phylogenetic tree of AGP data into 10 groups based on the co-phenetic distance matrix using the PAM algorithm. Then, we randomly selected two groups. If the total number of OTUs in the selected groups was greater than 17, we would stop. Otherwise, we would continue selecting the group randomly without replacement until there are ≥17 OTUs in the selected groups. Lastly, we pooled all the OTUs in the selected groups together and randomly assigned 17 OTUs as trait-associated. As the PAM algorithm partitions OTUs based on their phylogenetic distances, the associated OTUs from the clusters are phylogenetically closer to each other. This procedure is believed to be more realistic to represent the situation when associated taxa are phylogenetically related [6, 12, 27, 30, 32]. Also note that the PAM clusters are only for assigning the associated taxa, and we used the original taxonomic structure from AGP data while implementing the massMap.

For those 17 associated taxa, we considered two scenarios of association. Under scenario 1, effects of associated taxa have the same sign but varied strength, with small (β_j_∼ uniform (0, 2)), modest (β_j_∼ uniform (0, 3)) or large effect sizes (β_j_∼ uniform (0, 4)). In contrast, the effect directions were mixed in scenario 2, i.e., β_j_∼ uniform (− 2, 2), uniform (− 3,3), or uniform(− 4, 4). As sensitivity studies, we generated data where only 5% OTUs are trait-associate. This represents the condition when a much smaller proportion of OTUs are associated with the outcome. We also simulated data when associated taxa spread among the taxonomic tree. We partitioned the phylogenetic tree into 50 small groups using the PAM algorithm. Then 10% trait-associated OTUs are selected from the 50 groups instead of from the 10 groups. In the sensitivity studies, we only consider scenario 1 for the binary outcome as an illustration. In each simulation, we generated *N*=200 subjects and control the FDR at 0.05. Two thousand independent replications are conducted for each setting. The *p* values at screening rank and the target rank are estimated based on 1 × 10^5^ permutations.

### Simulation results

**Fig. 2:**
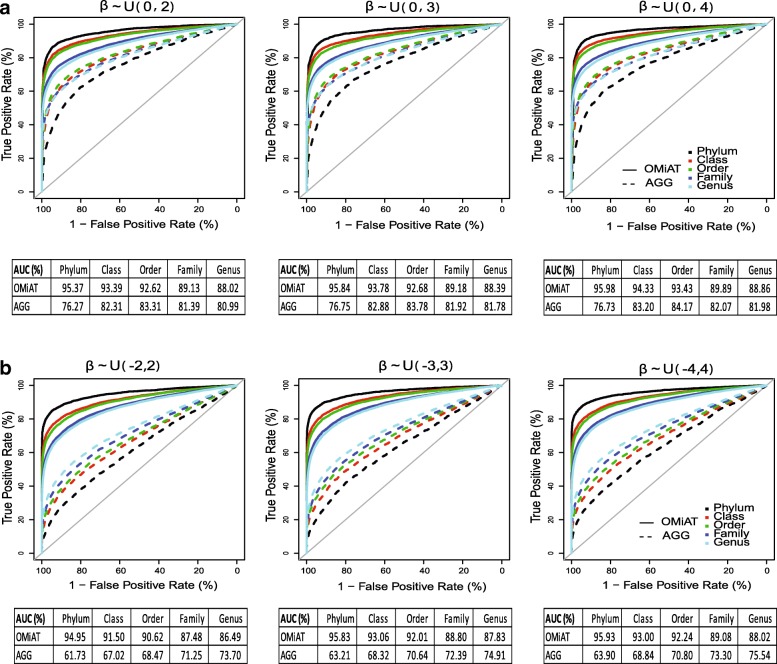
The ROC curves and area under the curves (AUCs) for OMiAT and the aggregated method for identifying the associated groups at the phylum, class, order, family, and genus ranks, respectively, in relation to the binary outcome variable. Panel **a** scenario 1: associated taxa have the same positive effect direction; **b** scenario 2: associated taxa have mixed-effect directions

In this section, we present the result for the binary outcome and defer the result for the continuous outcome in Additional files 1, 2 and 3: Figures S1–S3. We first evaluate the screening performance of OMiAT and the aggregated method using the receiver operating characteristic (ROC) curves and the area under the curves (AUCs) at the phylum, class, order, family, and genus ranks, respectively (Fig. 2). From the ROC, it is evident that OMiAT’s curves are consistently higher than those from the aggregated method for all ranks under both scenarios (Fig. 2a for the same effect direction and Fig. 2b for mixed directions). When we look into the AUC, we observe that OMiAT’s performance as a screening test is consistent between two scenarios and its AUC is highest at the phylum rank, and decreases as the taxonomic rank descends. This is explainable since OMiAT is more powerful when the group size is larger and the upper rank (such as phylum) groups consist of more target level taxa than do the lower rank groups. In contrast, the screening performance of the aggregated method is less satisfactory when the associated taxa within the testing group are more in mixed directions (Fig. 2b) than in the same direction (Fig. 2a), because the aggregation cancels the mixed-effect signals. Further, unlike OMiAT, the AUC of the aggregated method increases as the taxonomic rank descends because in theory the aggregated method achieves the highest power when all taxa within the group have the same effect size and direction. As groups become smaller at lower taxonomic ranks, the taxa within the group are more homogeneous, which increases the power of the aggregated method. In summary, through simulations, we verified that the aggregated method is not optimal for screening microbial associated groups at the upper rank. As expected, OMiAT exhibits marked performance as screening test statistics in the proposed two-stage framework.

**Fig. 3:**
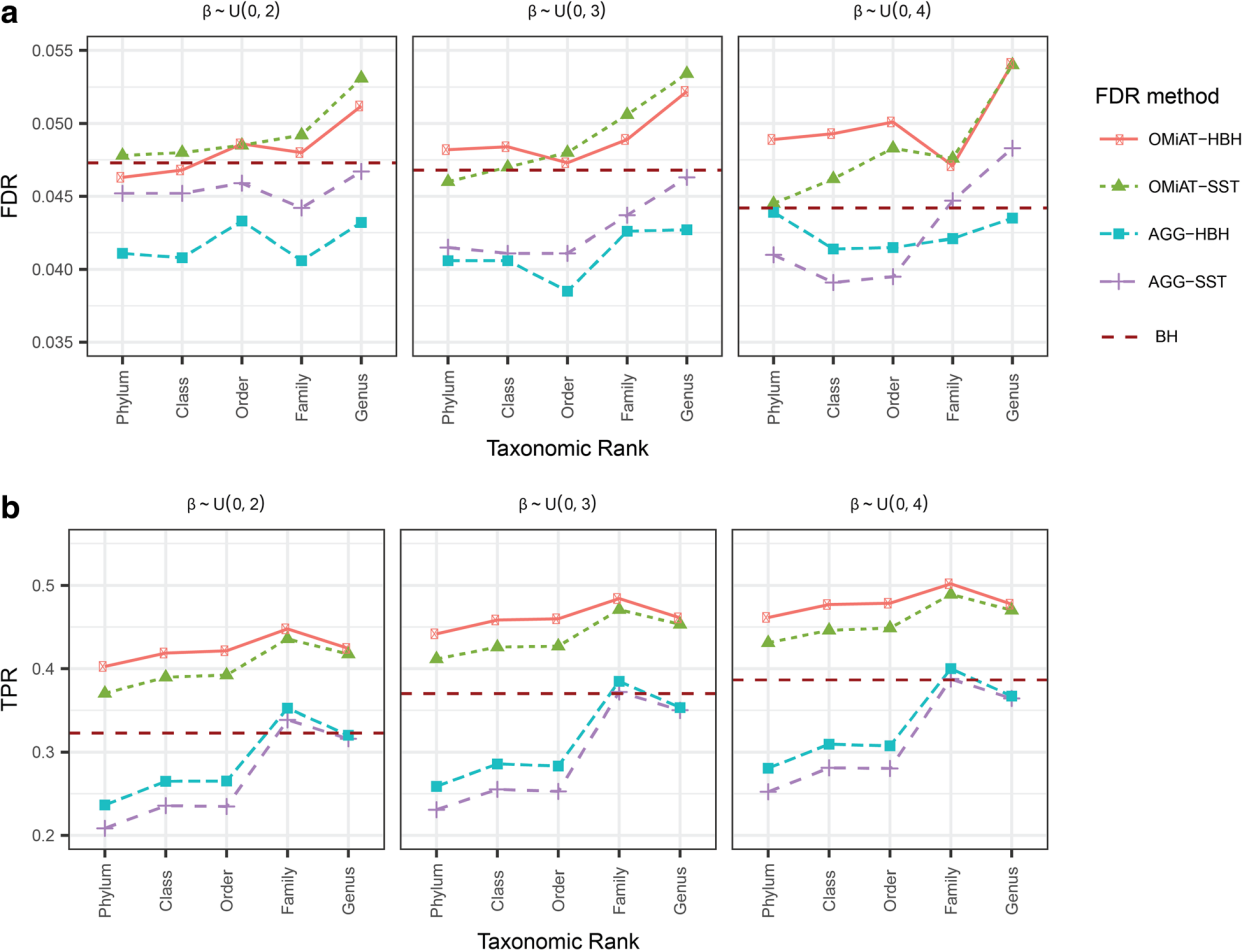
The false discovery rate (**a**) and true positive rate (power) (**b**) of massMap and the traditional BH method for the binary outcome variable. Scenario 1: the associated taxa have the same effect direction, with small (β ∼ uniform(0, 2), left panel), modest (β ∼ uniform(0, 3), middle panel), and large effect size (β ∼ uniform(0, 4), right panel)

**Fig. 4:**
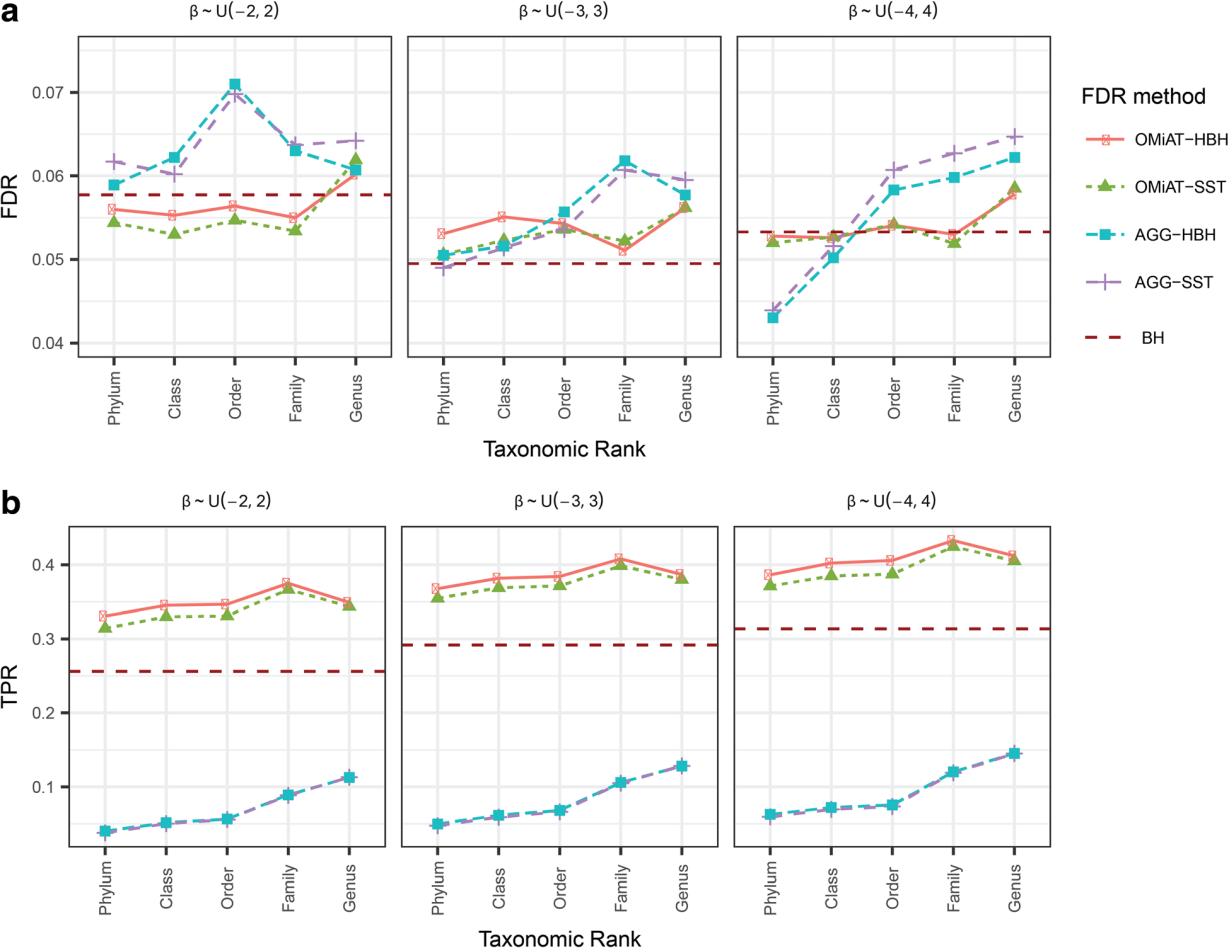
The false discovery rate (**a**) and true positive rate (power) (**b**) of massMap and the traditional BH method for the binary outcome variable. Scenario 2: the associated taxa have mixed effect directions, with small (β ∼ uniform(−2, 2), left panel), modest (β ∼ uniform(−3, 3), middle panel), and large effect size (β ∼ uniform(−4, 4), right panel)

Figures 3 and 4 report the empirical FDR and the TPR at the target rank for OMiAT-HBH, OMiAT-SST, AGG-HBH, and AGG-SST two-stage tests, when their screening tests are conducted at different taxonomic ranks for two association scenarios, respectively. The traditional one-stage method with BH procedure is considered as the benchmark in this simulation. The empirical FDR is defined as the proportion of the false discoveries among all discoveries at the target rank. From Figs. 3a and 4a, we observe that OMiAT-HBH and OMiAT-SST’s empirical FDRs are well-controlled around the nominal level 0.05, except when the screening test is conducted at genus rank in Scenario 2 with small effect size (empirical FDR = 0.064 and 0.062 for OMiAT-HBH and OMiAT-SST, respectively). Both AGG-HBH and AGG-SST can control the empirical FDR when the associated taxa are in the same direction (Fig. 3a), but they are susceptible to having inflated FDRs when the associated taxa are in mixed directions especially at the lower rank (Fig. 4a).

The TPR (power) is defined as the proportion of true positives among the true associated taxa at the target rank. Both AGG-HBH and AGG-SST suffer from poor power in both scenarios, owing to the inferior screening performance of the aggregated method. So we just focus on comparing the two-stage OMiAT-HBH and OMiAT-SST with the traditional BH method in the following. The proposed two-stage framework using OMiAT as the screening test has substantial power gain against the traditional BH method, no matter which rank is selected as the screening rank (Figs 3b and 4b). Noticeably, both OMiAT-HBH and OMiAT-SST reach the highest power when family rank is selected as the screening rank. This is a result of the balance between the screening test’s power and proportion of truly associated taxa among the significant groups. Comparing the results of HBH and SST for controlling FDR, we can see that HBH has relatively higher power than SST, since at the target rank, HBH corrects for the multiple comparisons within each discovered group. In comparison, SST pools candidate taxa from discovered groups together, leading to a higher penalty for multiple comparisons. When the rank of family is chosen as the screening stage, the results for HBH and SST are similar.

The result for the continuous outcome is consistent with that for the binary outcome, as shown in Additional files 1, 2 and 3: Figures S1-S3. Results of sensitivity studies show that OMiAT-HBH/OMiAT-SST has the FDR well controlled around the nominal level and has marked improvement on statistical power compared with BH and AGG-HBH/AGG-SST, when a much smaller proportion of OTUs are associated with the outcome (Additional file 4: Figure S4) and when associated taxa spread among the taxonomic tree (Additional file 5: Figure S5). In summary, two implementations of massMap, i.e., OMiAT-HBH and OMiAT-SST control the FDR around the nominal level for most of the simulation scenarios. They achieve substantial statistical power gain over the aggregated method based on the two-stage method and the traditional one-stage method, as they successfully incorporate the dependence structure into the microbial association mapping; their statistical powers peak when the screening is performed at the middle taxonomic rank family. Based on the simulation results, we recommend conducting the screening at the family rank using massMap.

### Real data applications

Here, we apply OMiAT-HBH, OMiAT-SST, AGG-HBH, AGG-SST, and traditional BH methods to the population-based American Gut Project (AGP) study and a murine gut microbiome dataset [28] to further compare their performance.

### American Gut Project

The American Gut Project (www.americangut.org) is a crowd-sourced project aimed at creating a comprehensive map of the human microbiome. The data include 16S rRNA V4 region sequences from 8610 fecal samples using Illumina MiSeq platform as well as the subjects’ metadata, as described in [33]. The full data set includes 22,891 OTUs from 7293 baseline samples. In our analyses, we excluded subjects who (1) were not USA resident; (2) had missing values in variables: sex, gender, body mass index (BMI), or antibiotic history (ABH); (3) were alcoholics; or (4) had BMI ≥ 80. After filtering, 1134 unique baseline samples were retained for analysis. Next, OTUs aligned to the bacteria kingdom were further filtered if (1) they could not be aligned to a family rank, or (2) they are presented in less than 3 individuals, or (3) average relative abundance <0.1%. After filtering, the abundance data related to 90 species were used for our analyses. We performed the microbial association mapping at the species rank on a binary outcome antibiotic history (ABH) and a continuous outcome body mass index (BMI), respectively. Sex and age are adjusted in both analyses.

#### Antibiotic history (ABH)

The ABH was coded into a binary response to indicate whether the subject had (ABH = 1, *n* = 761) or had not had (ABH = 0, *n* = 373) antibiotic usage in the preceding year. The logistic regression model in Eq. (1) was used to fit the data. As suggested by the simulation results, family rank was selected to conduct the screening test. With FDR = 0.05, 12 family groups (consisting of 37 species) were significantly associated with ABH by OMiAT, while only four groups (consisting of 5 species) are reported by the aggregated method (Additional file 6: Table S1). This indicates that OMiAT is much more powerful as a screening test compared with the aggregated method.

**Fig. 5:**
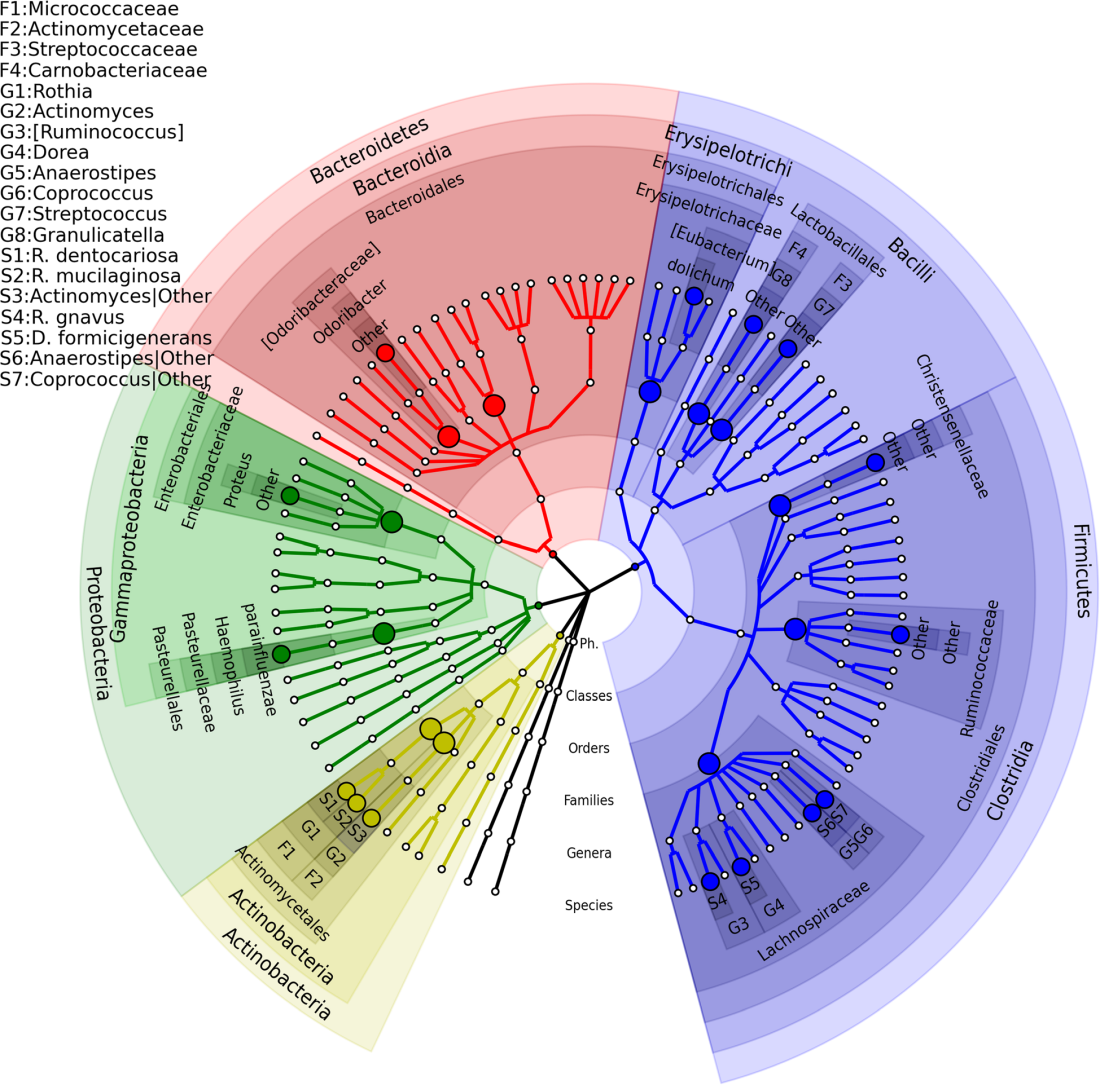
The microbial association mapping of antibiotic history (ABH) in American Gut Project subjects (FDR = 0.05). ABH-associated species with corresponding significant taxonomic groups reported by OMiAT-HBH and OMiAT-SST are highlighted. The taxonomic tree was generated using GraPhlAn [46]. The nodes on the tree from inner to outer circles are the phylum, class, order, family, genus, and species rank. The corresponding annotations are written in the reverse order. Family rank is selected to conduct the screening test for massMap

OMiAT-HBH, OMiAT-SST, and traditional BH methods each identified 15 ABH-associated species, among which 14 species are common (Table 1). In contrast, AGG-HBH and AGG-SST only identified five ABH associated species due to the miss-hits of the aggregated method at the screening rank. One possible reason that OMiAT-HBH/OMiAT-SST and the traditional BH method had similar performance was that the antibiotic effect was sufficiently strong to pass the stringent multiple comparison corrections in the traditional BH method in this study. However, OMiAT-HBH and OMiAT-SST produced much smaller FDR-adjusted *p* values than did the traditional BH method, which implies that OMiAT-HBH and OMiAT-SST are more efficient. The microbial association mapping results of OMiAT-HBH/OMiAT-SST are illustrated on a taxonomic tree (Fig. 5). There are four ABH associated microbial species clustered in family *Lachnospiraceae* and two species clustered in family M*icrococcaceae*. These observations are consistent with the hypothesis that evolutionarily closer taxa usually have similar responses to exposures. Additional file 7: Figure S6 shows the relative abundance distributions for discovered species. *[Ruminococcus] gnavus*, *Proteus|Other*, *Rothia mucilaginosa*, *Streptococcus|Other*, *Actinomyces|Other*, *[Eubacterium] dolichum*, *Rothia dentocariosa*, *Granulicatella|Other*, and *Haemophilus parainfluenzae* are more abundant in ABH = 1 group; and *Ruminococcaceae|Other*, *Odoribacter|Other*, *Christensenellaceae|Other*, *Anaerostipes|Other*, *Coprococcus|Other*, and *Dorea formicigenerans* are more abundant in ABH = 0 group.

**Table 1:** The raw and FDR-adjusted *p* values for the detected ABH-associated species using the AGP data (FDR = 0.05). Adjusted *p* values ≥0.05 are left blank

OTU ID	Species	Raw *p* value	BH	OMiAT-HBH	OMiAT-SST	AGG-HBH	AGG-SST
132657	*Ruminococcaceae|Other*	< 2.0E-06	1.80E-04	1.00E-05	7.40E-05		
164719	*[Ruminococcus] gnavus*	9.40E-05	4.20E-03	1.10E-03	1.70E-03		
4385479	*Proteus|Other*	2.90E-04	8.80E-03	1.20E-03	3.60E-03		
4476877	*Odoribacter|Other*	5.60E-04	1.30E-02	1.10E-03	5.20E-03		
903426	*Rothia mucilaginosa*	7.70E-04	1.40E-02	1.50E-03	5.70E-03	1.50E-03	3.20E-03
145236	*Christensenellaceae|Other*	1.30E-03	1.90E-02	1.30E-03	7.90E-03	1.30E-03	3.20E-03
1005952	*Streptococcus|Other*	3.40E-03	3.10E-02	3.40E-03	1.40E-02	3.40E-03	4.30E-03
4463237	*Actinomyces|Other*	3.40E-03	3.10E-02	3.40E-03	1.40E-02	3.40E-03	4.30E-03
177828	*Anaerostipes|Other*	3.50E-03	3.10E-02	2.10E-02	1.40E-02		
4328910	*Veillonella parvula*	3.50E-03	3.10E-02				
4396877	*[Eubacterium] dolichum*	4.30E-03	3.50E-02	1.70E-02	1.60E-02		
4466006	*Rothia dentocariosa*	5.00E-03	3.80E-02	5.00E-03	1.70E-02	5.00E-03	5.00E-03
47862	*Coprococcus|Other*	7.90E-03	4.90E-02	3.20E-02	2.20E-02		
1102921	*Granulicatella|Other*	8.10E-03	4.90E-02	8.10E-03	2.20E-02		
2654263	*Haemophilus parainfluenzae*	8.20E-03	4.90E-02	8.20E-03	2.20E-02		
4424063	*Dorea formicigenerans*	1.50E-02		4.40E-02	3.60E-02		
Number of detected ABH-associated species			15	15	15	5	5

#### Body mass index (BMI)

**Fig. 6:**
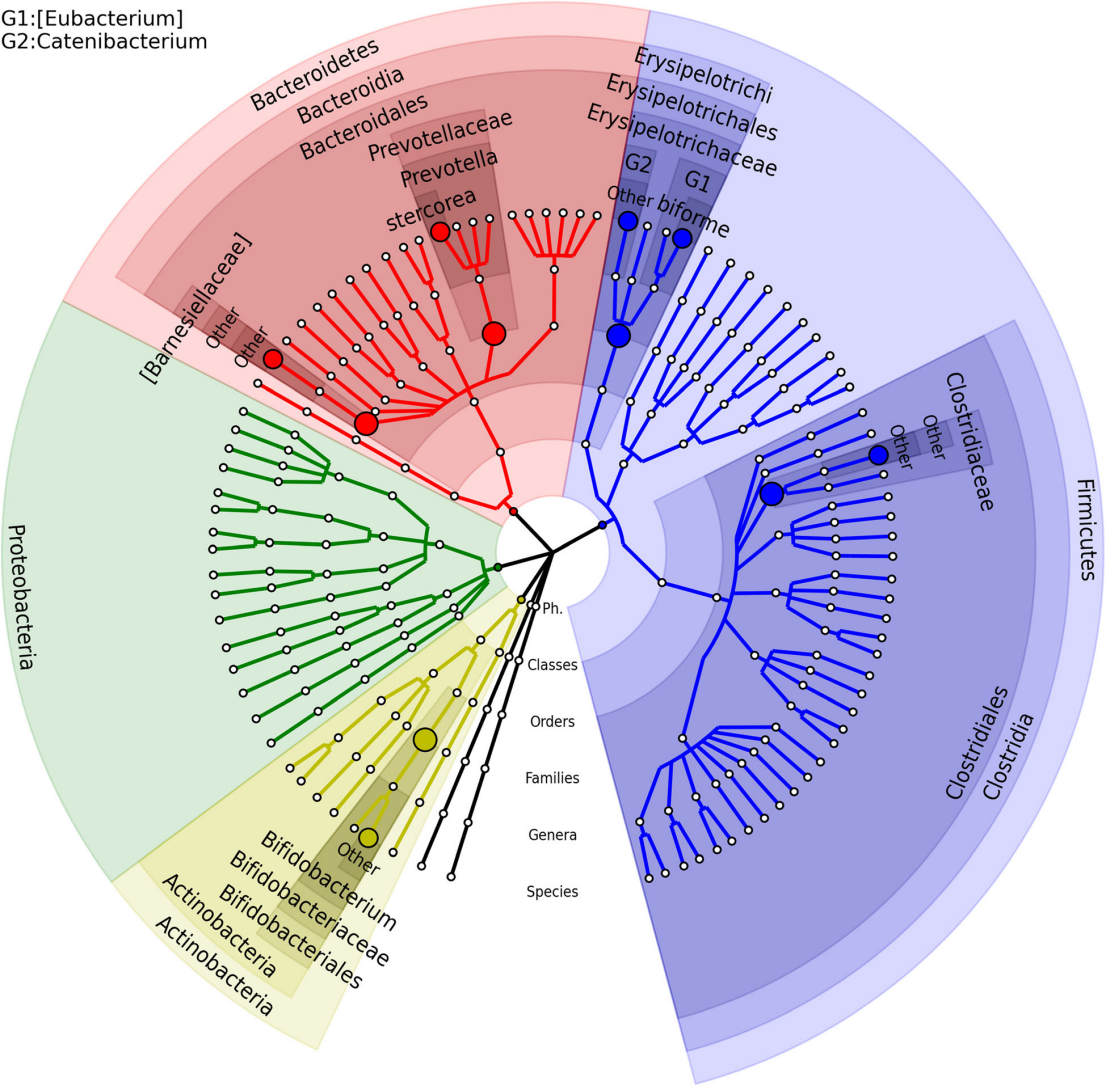
The microbial association mapping of body mass index (BMI) in American Gut Project subjects (FDR = 0.10). The associated species with corresponding significant taxonomic groups identified by OMiAT-HBH and OMiAT-SST are highlighted in the taxonomic tree. Among six detected species, *[Eubacterium] biforme* in phylum *Firmicutes* is also detected by the traditional BH method. The taxonomic tree was generated using GraPhlAn [46]. The nodes on the tree from inner to outer circles are the phylum, class, order, family, genus, and species rank. The corresponding annotations are written in the reverse order. Family rank is selected to conduct the screening test for massMap

The linear regression model in Eq. (2) was adopted to investigate the association between BMI for the AGP subjects and microbes at the species level. In massMap, as suggested by the simulations, screening tests were first performed at the family rank. With FDR = 0.10, 5 family groups (consisting of 13 species) were identified as associated with BMI by OMiAT, whereas none was identified by the aggregated method (Additional file 8: Table S2). Considering the target species level, the traditional BH, OMiAT-HBH, OMiAT-SST, AGG-HBH, and AGG-SST methods detected 1, 6, 6, 0, and 0 significant taxa associated with BMI respectively. The associated species identified by OMiAT-HBH/OMiAT-SST are further presented in the taxonomic tree in Fig. 6. Compared with the traditional BH and massMap with the aggregated method, OMiAT-HBH and OMiAT-SST discover more taxa and exhibit extra power gain, consistent with the simulations.

The OMiAT-HBH/OMiAT-SST identified species: *[Eubacterium] biforme*, *Bifidobacterium|Other*, *Catenibacterium|Other*, and *Prevotella stercorea* (bold highlighted in Table 2) were also reported to be associated with BMI or related phenotypes in other studies [34–36]. Our results show that the abundances of *[Eubacterium] biforme*, *Catenibacterium|Other*, and *Prevotella stercorea* were positively associated with BMI; and the abundance of *Bifidobacterium|Other* was negatively associated with BMI (Additional file 9: Figure S7). In two separate studies [33, 34], genus *Bifidobacterium* was present at higher abundance in normal-weight than in overweight women. In another study, two *Bifidobacterium* strains have significant effects on obesity in high-fat diet induced rats [34]. Zhang et al. (2008) [35] reported that the *Erysipelotrichaceae* (from phylum *Firmicutes*) and *Prevotellaceae* (from phylum *Bacteroidetes*) families are more abundant in the obesity group than in the normal weight group. In our analyses, identifying microbial associations at the species level, *Catenibacterium|Other* with OTU ID 4480861 from family *Erysipelotrichaceae*, and *Prevotella stercorea* with OTU ID 513664 from family *Prevotellaceae* were positively associated with BMI.

**Table 2:** The raw and FDR-adjusted *p* values for the detected BMI-associated species using the AGP data (FDR = 0.10). Adjusted *p* values ≥0.10 are left blank. Note that there is no associated species reported by the aggregated method. The biological evidence for the bold highlighted taxa has been found in the literature [34–36] and discussed in the text

OTU ID	Species	Raw p-value	BH	OMiAT-HBH	OMiAT-SST
297635	***[Eubacterium] biforme***	1.90E-04	1.70E-02	7.60E-04	2.50E-03
824876	***Bifidobacterium|Other***	2.70E-03		5.30E-03	1.70E-02
4319938	*Clostridiaceae|Other*	1.00E-02		2.00E-02	3.50E-02
840279	*[Barnesiellaceae]|Other*	1.10E-02		1.10E-02	3.50E-02
4480861	***Catenibacterium|Other***	1.50E-02		3.10E-02	4.00E-02
513664	***Prevotella stercorea***	2.00E-02		8.00E-02	4.30E-02
Number of detected BMI-associated species			1	6	6

As a sensitivity analysis, the traditional BH, OMiAT-HBH, OMiAT-SST, AGG-HBH, and AGG-SST methods detected 1, 3, 3, 0, and 0 significant species associated with BMI respectively with FDR = 0.05 (Additional file 10: Table S3). All methods identified fewer species when a more stringent FDR threshold was used, but OMiAT-HBH and OMiAT-SST discovered more.

### The effects of early-life antibiotics on murine intestinal microbiota

Livanos et al. (2016) [28] has conducted a longitudinal microbiome study to examine whether early-life sub-therapeutic antibiotic treatment (STAT) would alter the gut microbiota and accelerate T1D onset in non-obese diabetic (NOD) mice. DNA samples from feces were analyzed by targeting the V4 region of the bacterial 16S rRNA genes as described in [37]. Using the QIIME pipeline [38], OTU table and phylogenetic tree were created for 28 control and 21 STAT male mice. Compared with the observational AGP data, the number of observed OTUs and the size of the phylogenetic tree are both much smaller in this experimental mouse study. We re-examined the antibiotic effect on the fecal microbiome immediately after weaning (week 3). Originally, 75 species were observed. After filtering species that were present in ≤ 3 mice, 36 were retained for analysis.

**Fig. 7:**
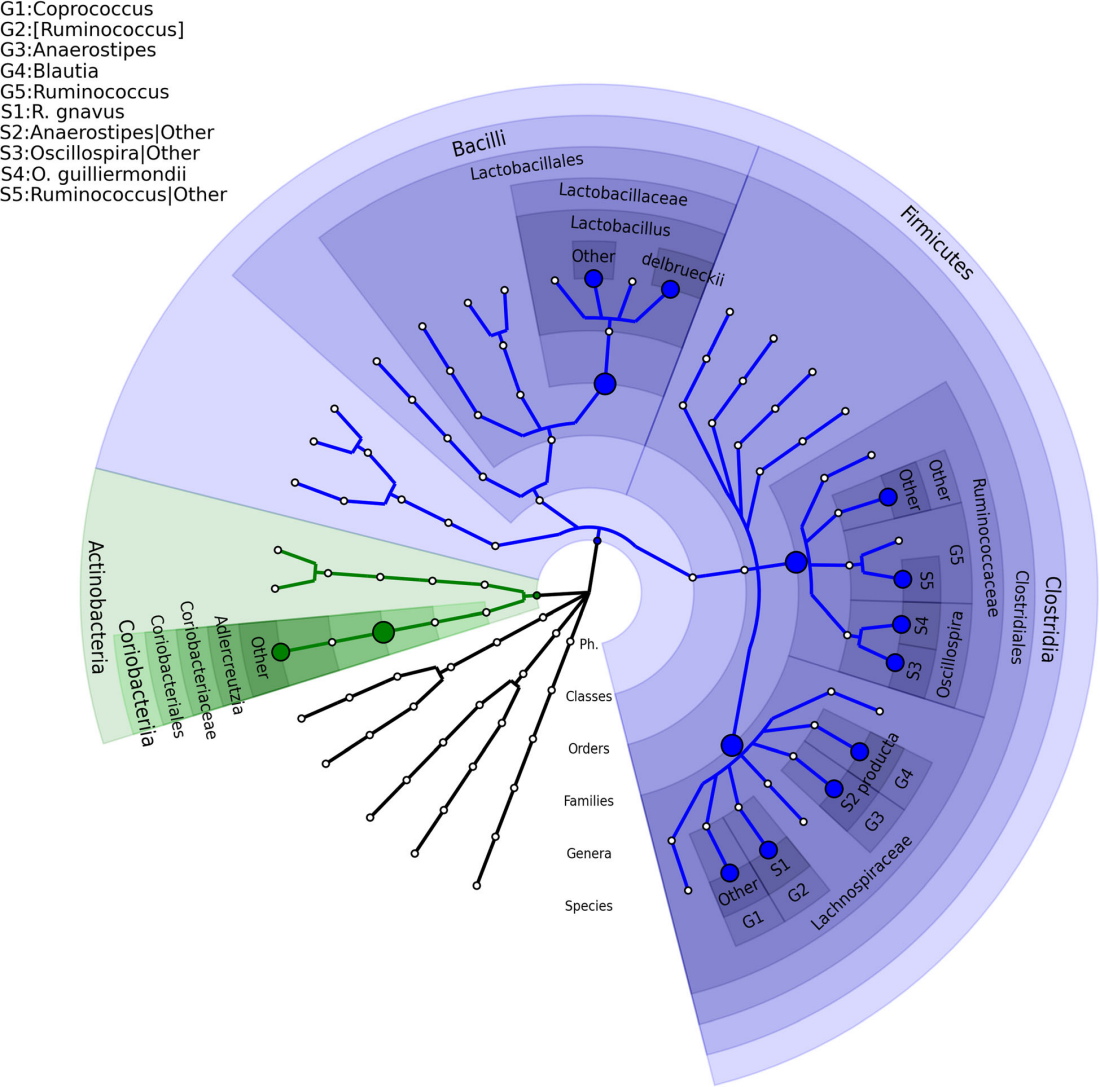
The microbial association mapping of STAT vs control groups using the murine data (FDR = 0.05). STAT-associated species with corresponding significant taxonomic groups reported by OMiAT-HBH and OMiAT-SST are highlighted. The taxonomic tree was generated using GraPhlAn [46]. The nodes on the tree from inner to outer circles are the phylum, class, order, family, genus, and species rank. The corresponding annotations are written in the reverse order. Family rank is selected to conduct the screening test for massMap

The logistic regression model in Eq. (1) was employed to detect differentially abundant species between the STAT and control groups. The screening test was conducted at the family rank in massMap. With FDR = 0.05, OMiAT and the aggregated method identified four and two groups associated with the antibiotic treatment, respectively (Additional file 11: Table S4). At the target species rank, the traditional BH, OMiAT-HBH, OMiAT-SST, AGG-HBH, and AGG-SST methods detected 9, 11, 11, 4, and 4 significant species associated with antibiotic treatment respectively (Table 3). massMap using OMiAT, i.e., OMiAT-HBH and OMiAT-SST, reported two more significant species than the traditional BH method, whereas the aggregated method had the fewest findings. Two species *Lactobacillus delbrueckii* and *Oscillospira guilliermondii* explicitly reported by OMiAT-HBH and OMiAT-SST have much lower relative abundances in STAT mice than in control mice (Additional file 12: Figure S8). The associated species reported by the OMiAT-HBH and OMiAT-SST are presented in the taxonomic tree (Fig. 7), and they indicate a clear clustering association pattern. From the figure, we observe that there are four associated species clustered in family *Lachnospiraceae* and four species clustered in family *Ruminococcaceae* that are differentially presented in STAT and control groups. With clustered signals, massMap achieves higher power as expected.

**Table 3:** The raw and FDR-adjusted *p* values for the detected STAT-associated species using the murine data (FDR = 0.05). Adjusted *p* values ≥0.05 are left blank

OTU ID	Species	Raw *p* value	BH	OMiAT-HBH	OMiAT-SST	AGG-HBH	AGG-SST
33701	*Adlercreutzia| Other*	2.90E-04	1.00E-02	2.90E-04	5.10E-03		
37580	*Lactobacillus| Other*	1.70E-03	2.40E-02	6.90E-03	1.20E-02		
9867	*Ruminococcaceae| Other*	2.00E-03	2.40E-02	9.20E-03	1.20E-02	9.20E-03	6.90E-03
50804	*Ruminococcus| Other*	3.70E-03	3.00E-02	9.20E-03	1.50E-02	9.20E-03	8.00E-03
47849	*Oscillospira| Other*	4.60E-03	3.00E-02	9.20E-03	1.50E-02	9.20E-03	8.00E-03
34428	*Coprococcus| Other*	5.40E-03	3.00E-02	1.30E-02	1.50E-02		
195457	*Blautia producta*	6.80E-03	3.00E-02	1.30E-02	1.50E-02		
35567	*[Ruminococcus] gnavus*	7.00E-03	3.00E-02	1.30E-02	1.50E-02		
2416	*Anaerostipes| Other*	7.60E-03	3.00E-02	1.30E-02	1.50E-02		
10675	*Lactobacillus delbrueckii*	1.70E-02		3.30E-02	3.00E-02		
20780	*Oscillospira guilliermondii*	2.30E-02		3.50E-02	3.80E-02	3.50E-02	3.20E-02
Number of detected STAT-associated species			9	11	11	4	4

## Discussion

massMap can easily be extended to examine survival outcome. A highly adaptive microbial group association test for survival outcome (OMiSA) has been separately developed by our group [39]. Upon publication, it will be added to our massMap software. Furthermore, the concepts underlying massMap can be generalized to the functional pathway analysis of metagenomics data. Confronting numerous pathways, one can first test for differential microbial abundance at the pathway level and then proceed to test for differential microbial abundance at the gene family level. In practice, to implement massMap, it is required to pre-determine the screening rank. Based on the simulation results and our practical experience with the real data analyses, we recommend conducting the screening at the middle taxonomic rank family. We should note that massMap is an association mapping tool and does not make causal inference. It provides candidate microbial taxa for the follow-up validation studies, where additional experiments could elucidate the causal role of the discovered taxa on the outcome trait.

massMap uses the relative abundance of taxa to perform the association analysis. The relative abundance data is compositional as the summation in each sample is constrained to one [40]. The log-ratio transformation of relative abundances have been used to relieve the compositionality issue [6] in various methods. However, since the compositional effect is attenuating as the number of taxa increases and our goal is to detect which taxa (instead of ratio of taxa) are associated with the outcome, we prefer to work on the relative abundance for better interpretation. A school of statistical methods working on the relative abundance data have been developed, including differential abundance analyses [41–43] and association mapping analyses [18, 27, 29].

We shed light on the usage of advanced FDR controlling methodologies [21–24] in the two-stage microbial association analysis. Our simulations were based on the large-scale population-level microbiome AGP study with 174 OTUs after filtering. The HBH and SST FDR-controlling procedures can control the FDR in most scenarios while among few scenarios, we did observe minor FDR inflation in HBH and/or SST procedures. Similar two-stage analysis has been studied in analyzing microarray data; when there are a large number of hypotheses (usually in the thousands) at the target level in microarray studies, an inflated FDR for the HBH procedure has been reported [21]. Reiner-Benaim et al. (2007) [25] presented a corrected threshold *q*^∗^ for both stages to control the FDR around the nominal level in the microarray data analysis setting. A similar correction can be considered if the number of hypotheses in the application is very large.

Sankaran and Holmes (2014) [44] applied the HBH procedure to an environmental microbiome dataset based on phylogenetic tree of the microbes, which is different from the proposed massMap based on the taxonomic tree. The phylogenetic tree is deeper than the taxonomic tree with complex structure. It consists of many levels and every parent node has a maximum of two daughter nodes. It is not clear whether the HBH procedure applied to the phylogenetic tree proposed in [38] can control FDRs or not. Xiao et al. (2017) [45] also proposed an FDR-controlling method based on the hierarchical tree (TreeFDR), with the underlying assumption that associated taxa have the same effect direction and are clustered in the phylogenetic tree. When this assumption is violated such as in scenario 2 of our simulation study, TreeFDR would suffer great power loss as the aggregated method did.

## Conclusions

In this paper, we focus on developing a two-stage microbial association mapping framework called massMap for binary and continuous outcomes. MassMap incorporates the highly powerful microbial group test OMiAT for screening and HBH/SST for the control of FDR. Compared with the traditional one-stage method, massMap achieves marked improvements in statistical power while well controlling FDR under most scenarios. Consequently, we recommend that massMap can be extensively used for microbiome-wide association analyses as a highly efficient method.

## Additional files

Additional file 1: **Figure S1.** The ROC curves and area under the ROC curves (AUCs) for OMiAT and the aggregated method for identifying the associated groups at the phylum, class, order, family, and genus ranks, respectively, in relation to the continuous outcome variable. Panel (A) scenario 1: analysis of associated taxa that have the same positive effect direction. (B) Scenario 2: analysis of associated taxa that have mixed effect directions. (PDF 13004 kb)

Additional file 2: **Figure S2.** The false discovery rate (A) and true positive rate (power) (B) of massMap and the traditional BH method for the continuous outcome variable. Scenario 1: the associated taxa have the same effect direction, with small (β ∼ uniform(0, 2), left panel), modest (β ∼ uniform(0, 3), middle panel), and large (β ∼ uniform(0, 4), right panel) effect sizes. (PNG 397 kb)

Additional file 3: **Figure S3.** The false discovery rate (A) and true positive rate (power) (B) of massMap and the traditional BH method for the continuous outcome variable. Scenario 2: the associated taxa have mixed effect directions, with small (β ∼ uniform(−2, 2), left panel), modest (β ∼ uniform(−3, 3), middle panel), and large (β ∼ uniform(−4, 4), right panel) effect sizes. (PNG 370 kb)

Additional file 4: **Figure S4.** The false discovery rate (A) and true positive rate (power) (B) of massMap and the traditional BH method for the binary outcome variable. Five percent OTUs are assigned as the truly associated taxa and have the same effect direction, with small (β ∼ uniform(0, 2), left panel), modest (β ∼ uniform(0, 3), middle panel), and large (β ∼ uniform(0, 4), right panel) effect sizes. (PNG 135 kb)

Additional file 5: **Figure S5.** The false discovery rate (A) and true positive rate (power) (B) of massMap and the traditional BH method for the binary outcome variable. We partitioned the phylogenetic tree into 50 groups using PAM algorithm. Ten percent OTUs are assigned as trait-associated, with small (β ∼ uniform(0, 2), left panel), modest (β ∼ uniform(0, 3), middle panel), and large (β ∼ uniform(0, 4), right panel) effect sizes. (PNG 140 kb)

Additional file 6: **Table S1.** Candidate taxonomic groups at the family rank associated with a history of recent antibiotic use (ABH) in the AGP data analysis. Groups were detected either by OMiAT or by the aggregate method, respectively. FDR = 0.05. (PDF 91 kb)

Additional file 7: **Figure S6.** Relative abundances of ABH-associated species present in the fecal samples from AGP subjects without (indicated as 0) or with (indicated as 1) recent antibiotic use (ABH). The species were detected by the proposed two-stage framework OMiAT-HBH and OMiAT-SST. The groups with/without recent antibiotic exposure had 761 and 373 subjects, respectively. (PDF 930 kb)

Additional file 8: **Table S2.** The family groups that were associated with BMI detected by OMiAT in AGP data (FDR = 0.10). The aggregated method did not identify any significant groups at the family rank. (PDF 143 kb)

Additional file 9: **Figure S7.** The relative abundances of BMI-associated species for AGP subjects grouped by their BMI quantile. The species shown had been detected by the proposed two-stage framework OMiAT-HBH and OMiAT-SST (FDR = 0.10). (PDF 309 kb)

Additional file 10: **Table S3.** The unadjusted and FDR-adjusted *p* values for the detected BMI-associated species using the AGP data (FDR = 0.05). Adjusted *p* values ≥0.05 are left blank. There were no associated species identified by the aggregated method. (PDF 155 kb)

Additional file 11: **Table S4.** Candidate groups (at the family rank) associated with STAT exposure, detected by OMiAT or by the aggregated method, respectively, using data from a murine experiment [28] (FDR = 0.05). (PDF 85 kb)

Additional file 12: **Figure S8.** Comparison of the relative abundances (RA) of the significant species between control (CONT) and STAT (STAT) groups. The species were detected by massMap: OMiAT-HBH and OMiAT-SST (FDR = 0.05). (PDF 1883 kb)

## Abbreviations

ABH: Antibiotic history
AGG: The aggregated method
AGP: American Gut Project
BH: Benjamini-Hochberg
BMI: Body mass index
DM: Dirichlet-multinomial
FDR: False discovery rate
HBH: Hierarchical BH
MiRKAT: Microbiome regression-based kernel association test
NOD mice: Non-obese diabetic mice
OMiAT: Optimal microbiome-based association test
OTU: Operational taxonomic unit
RA: Relative abundance
SPU: Sum of score powered tests
SST: Selected subset testing
STAT: Sub-therapeutic antibiotic treatment
T1D: Type 1 diabetes
TPR: True positive rate
