# Supplementary material for: A two-stage microbial association mapping framework with advanced FDR control
Source: Microbiome. 2018 Jul 25;6:131. doi: 10.1186/s40168-018-0517-1 (PMC6060480; doi:10.1186/s40168-018-0517-1)
Supplement: Supplementary file 1 — Figure S1. The ROC curves and area under the ROC curves (AUCs) for OMiAT and the aggregated method for identifying the associated groups at the phylum, class, order, family, and genus ranks, respectively, in relation to the continuous outcome variable. Panel (A) scenario 1: analysis of associated taxa that have the same positive effect direction. (B) Scenario 2: analysis of associated taxa that have mixed effect directions. (PDF 13004 kb) [file 40168_2018_517_MOESM1_ESM.pdf]

(A)

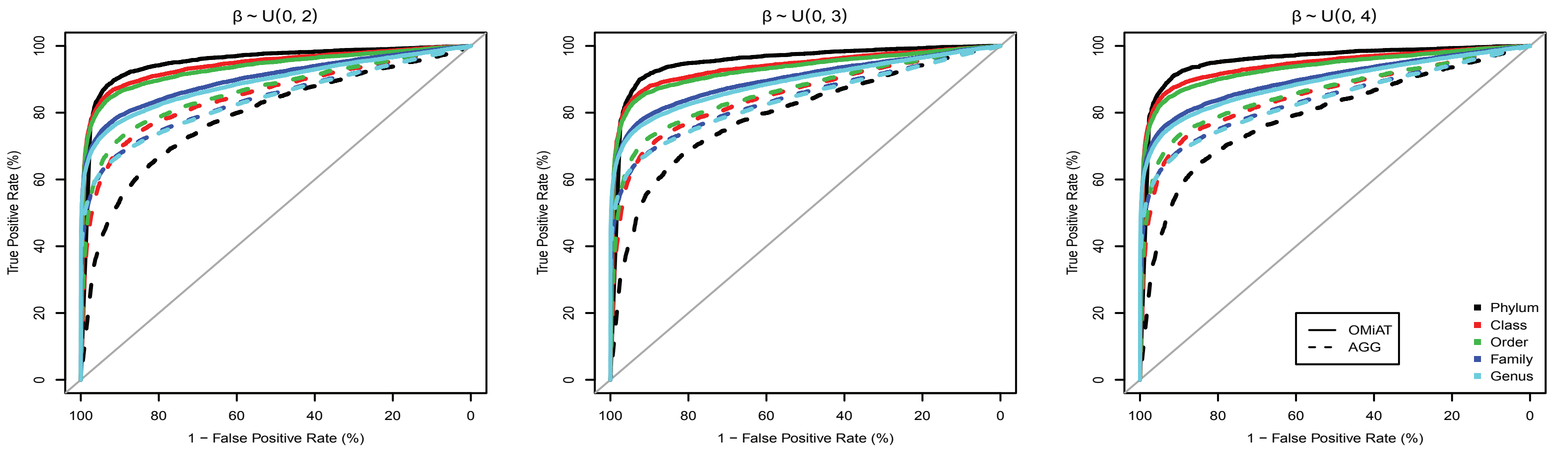

AUC (%)	Phylum	Class	Order	Family	Genus
OMiAT	95.11	94.08	93.27	90.02	88.95
AGG	78.90	84.86	86.13	83.76	83.64

AUC (%)	Phylum	Class	Order	Family	Genus
OMiAT	95.26	93.57	92.88	89.85	89.01
AGG	79.48	85.01	86.02	84.00	83.75

AUC (%)	Phylum	Class	Order	Family	Genus
OMiAT	95.57	94.15	93.29	89.89	88.96
AGG	79.08	85.05	85.93	83.90	83.72

(B)

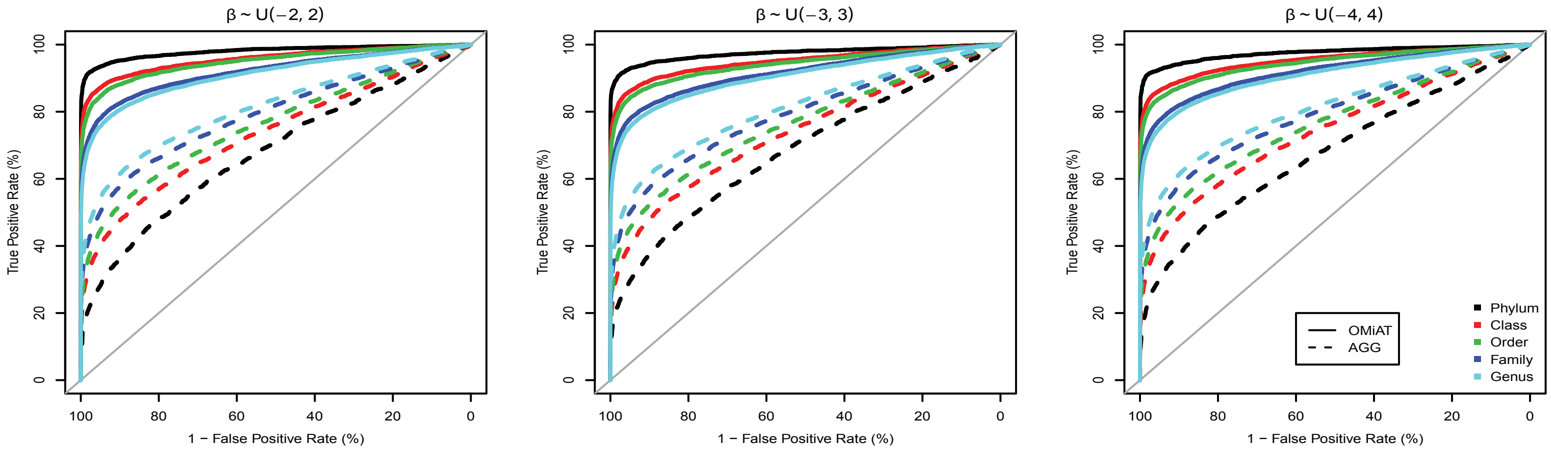

AUC (%)	Phylum	Class	Order	Family	Genus
OMiAT	97.94	95.58	94.81	91.89	91.05
AGG	67.95	73.42	75.94	79.08	81.13

AUC (%)	Phylum	Class	Order	Family	Genus
OMiAT	97.40	94.89	94.00	91.46	90.53
AGG	68.43	73.70	76.02	78.87	80.91

AUC (%)	Phylum	Class	Order	Family	Genus
OMiAT	97.48	95.17	94.39	91.85	90.89
AGG	67.86	74.14	76.38	79.10	80.90
