# Supplementary figures and images for: A two-stage microbial association mapping framework with advanced FDR control
Source: Microbiome. 2018 Jul 25;6:131. doi: 10.1186/s40168-018-0517-1 (PMC6060480; doi:10.1186/s40168-018-0517-1)

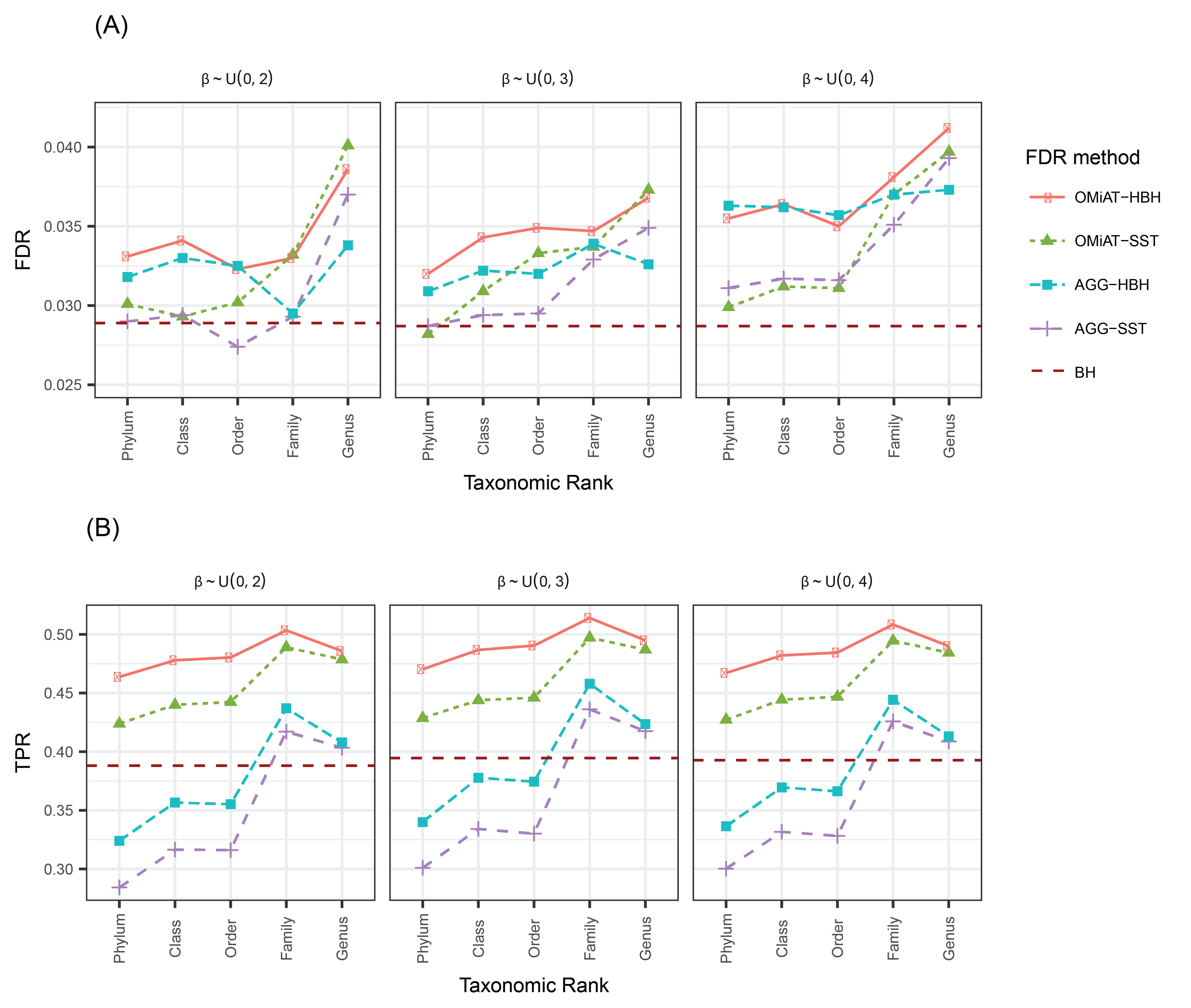

Supplement: Supplementary file 2 — Figure S2. The false discovery rate (A) and true positive rate (power) (B) of massMap and the traditional BH method for the continuous outcome variable. Scenario 1: the associated taxa have the same effect direction, with small (β ∼ uniform(0, 2), left panel), modest (β ∼ uniform(0, 3), middle panel), and large (β ∼ uniform(0, 4), right panel) effect sizes. (PNG 397 kb) [file 40168_2018_517_MOESM2_ESM.png]

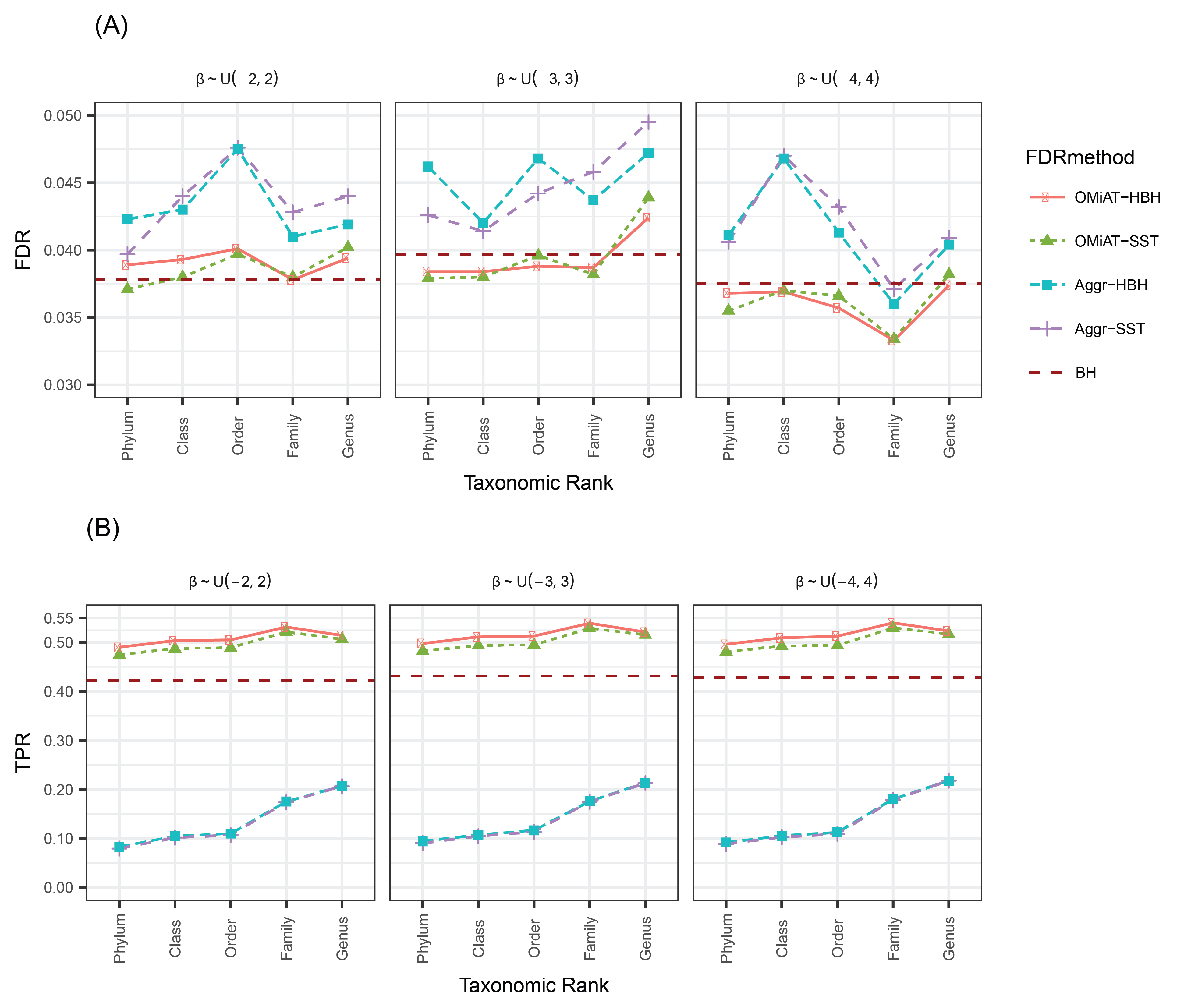

Supplement: Supplementary file 3 — Figure S3. The false discovery rate (A) and true positive rate (power) (B) of massMap and the traditional BH method for the continuous outcome variable. Scenario 2: the associated taxa have mixed effect directions, with small (β ∼ uniform(−2, 2), left panel), modest (β ∼ uniform(−3, 3), middle panel), and large (β ∼ uniform(−4, 4), right panel) effect sizes. (PNG 370 kb) [file 40168_2018_517_MOESM3_ESM.png]

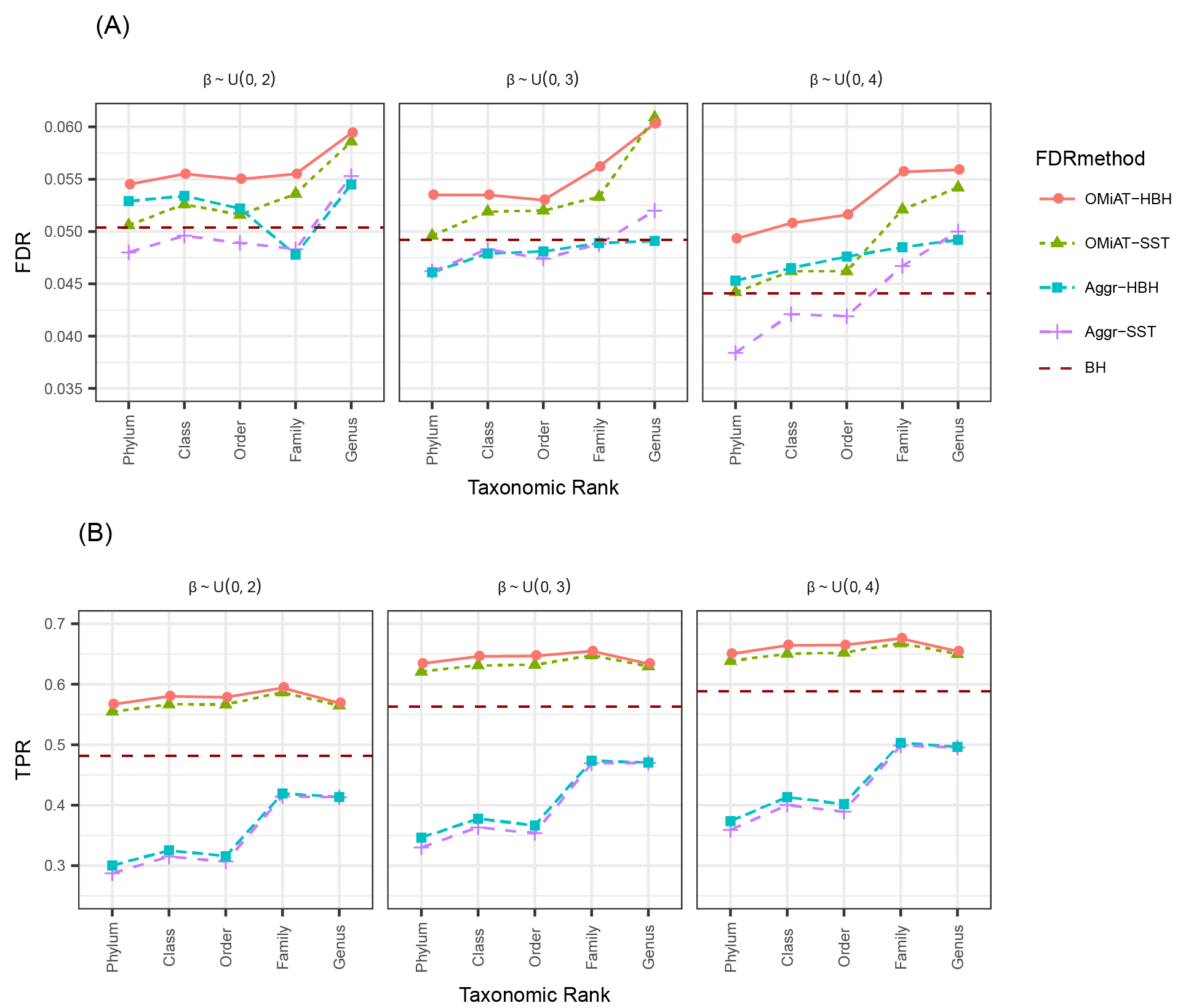

Supplement: Supplementary file 4 — Figure S4. The false discovery rate (A) and true positive rate (power) (B) of massMap and the traditional BH method for the binary outcome variable. Five percent OTUs are assigned as the truly associated taxa and have the same effect direction, with small (β ∼ uniform(0, 2), left panel), modest (β ∼ uniform(0, 3), middle panel), and large (β ∼ uniform(0, 4), right panel) effect sizes. (PNG 135 kb) [file 40168_2018_517_MOESM4_ESM.png]

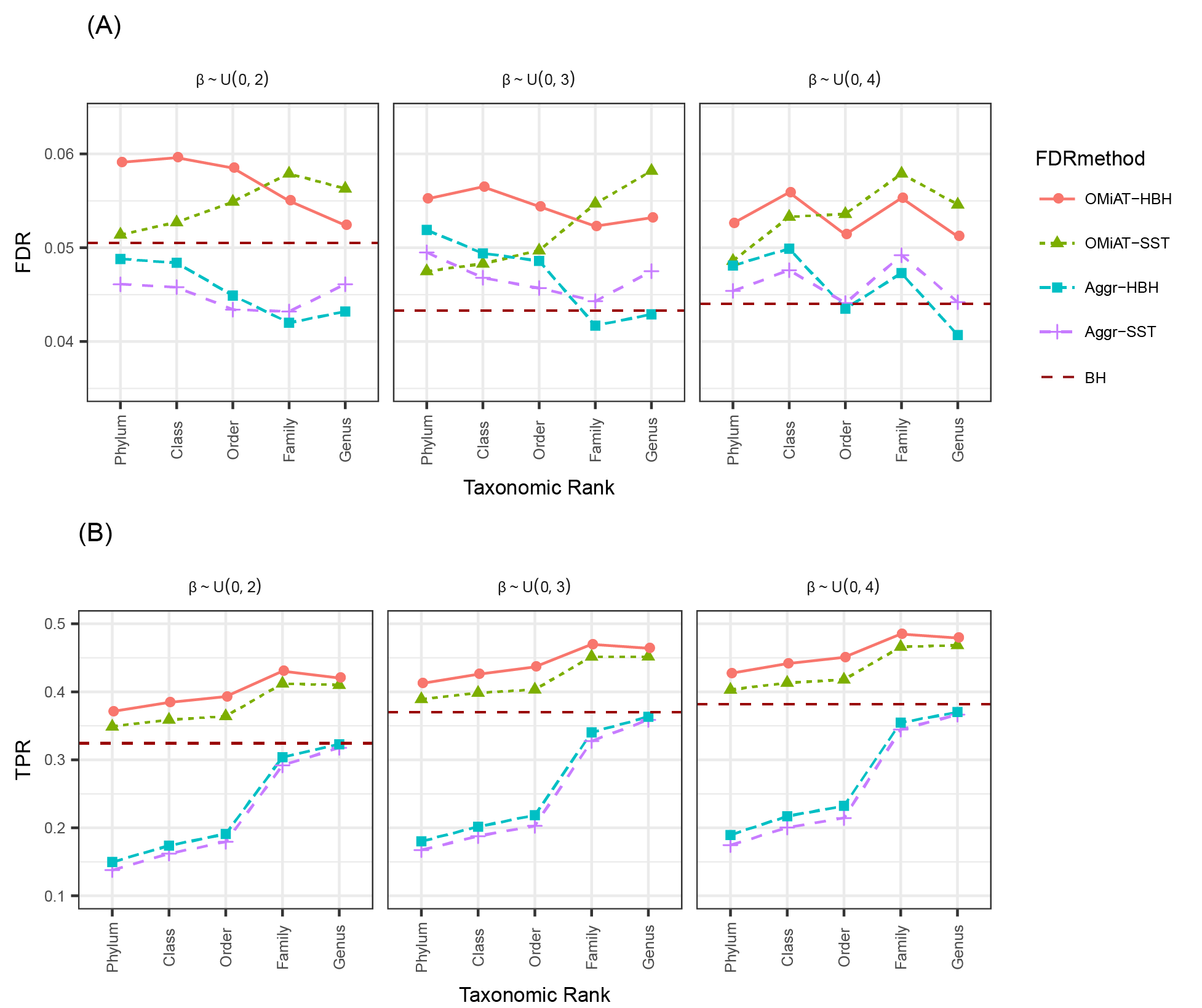

Supplement: Supplementary file 5 — Figure S5. The false discovery rate (A) and true positive rate (power) (B) of massMap and the traditional BH method for the binary outcome variable. We partitioned the phylogenetic tree into 50 groups using PAM algorithm. Ten percent OTUs are assigned as trait-associated, with small (β ∼ uniform(0, 2), left panel), modest (β ∼ uniform(0, 3), middle panel), and large (β ∼ uniform(0, 4), right panel) effect sizes. (PNG 140 kb) [file 40168_2018_517_MOESM5_ESM.png]

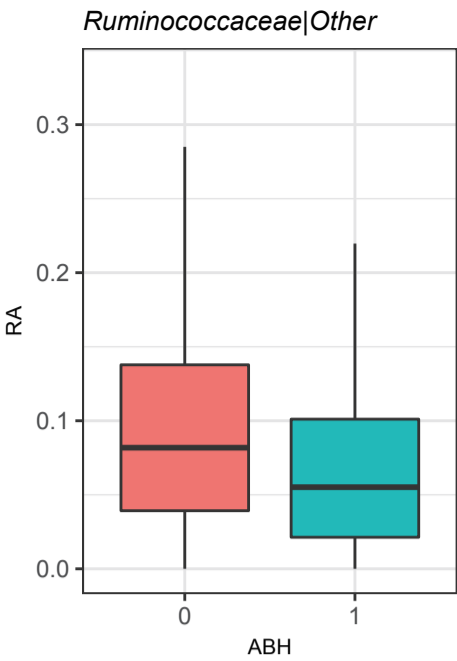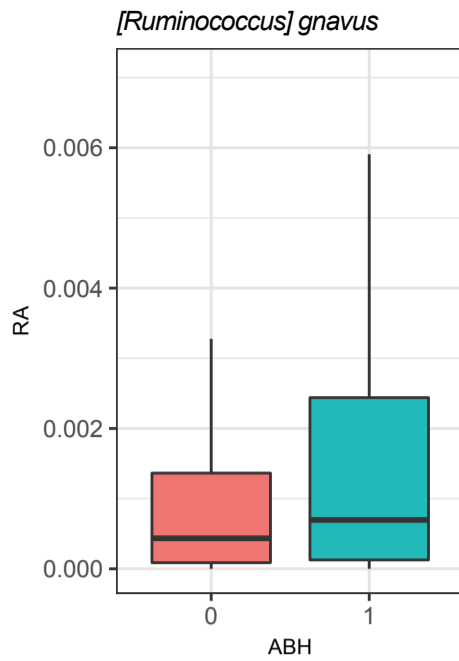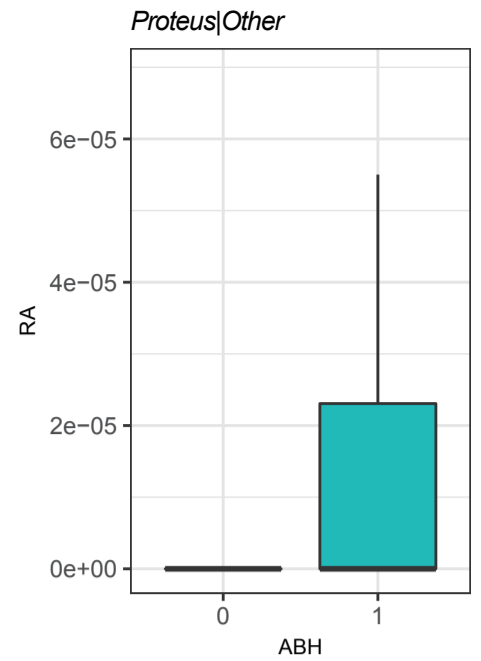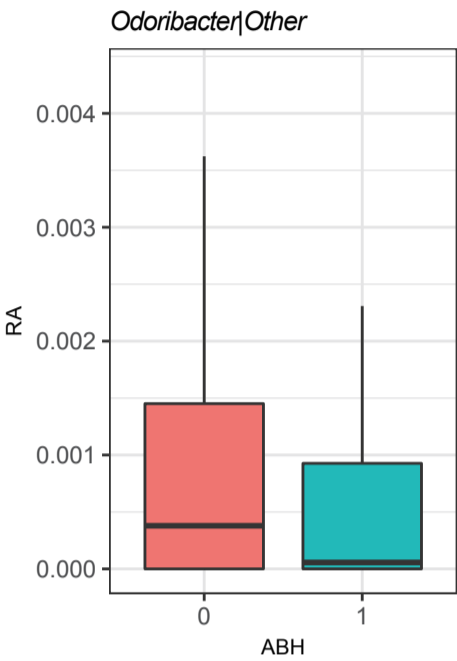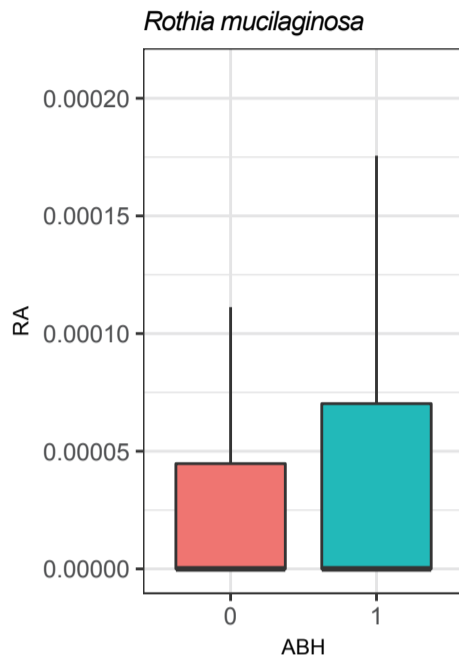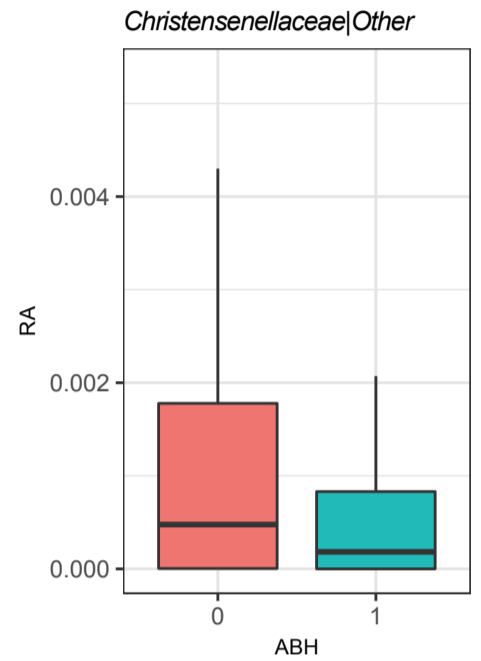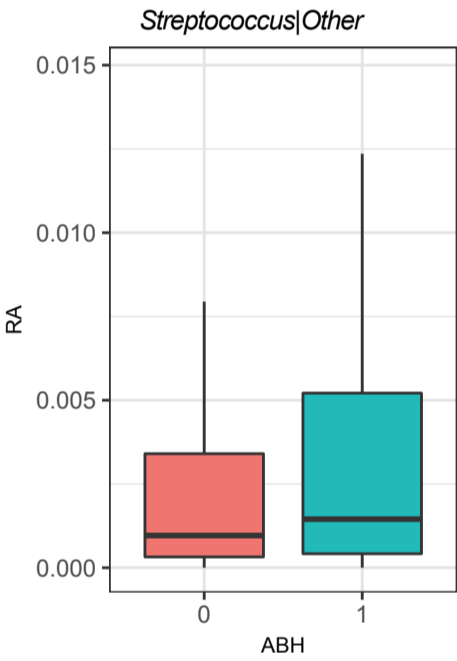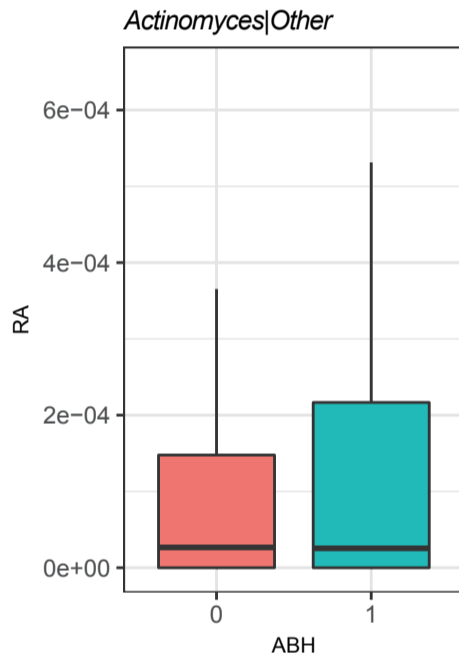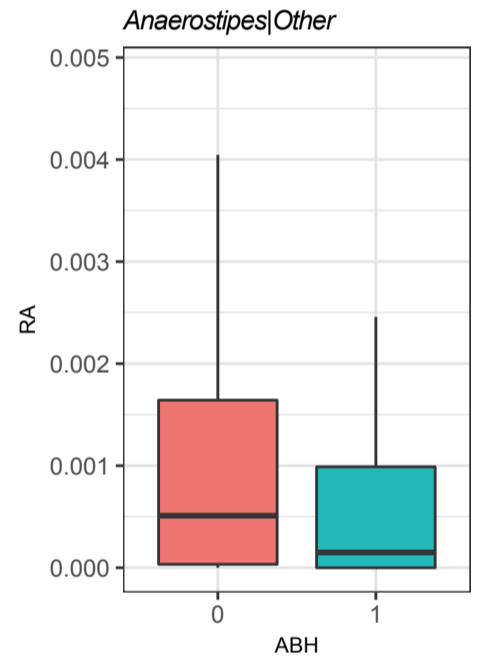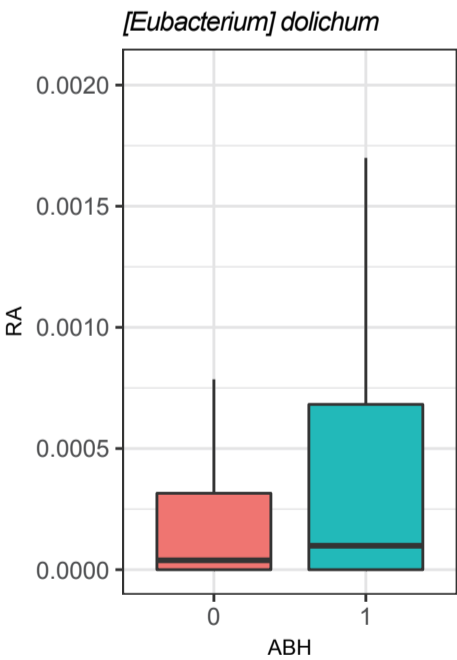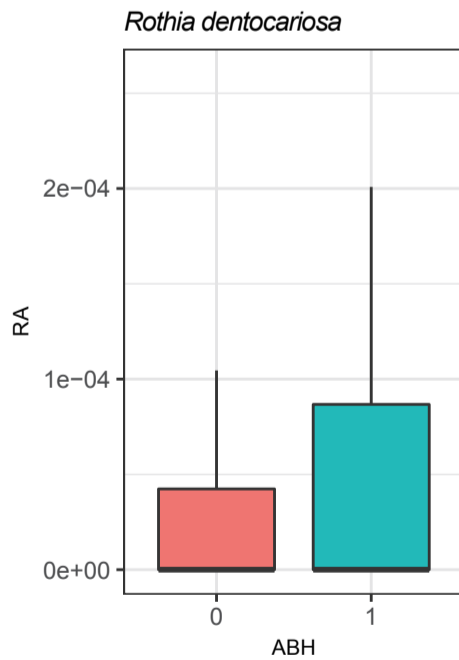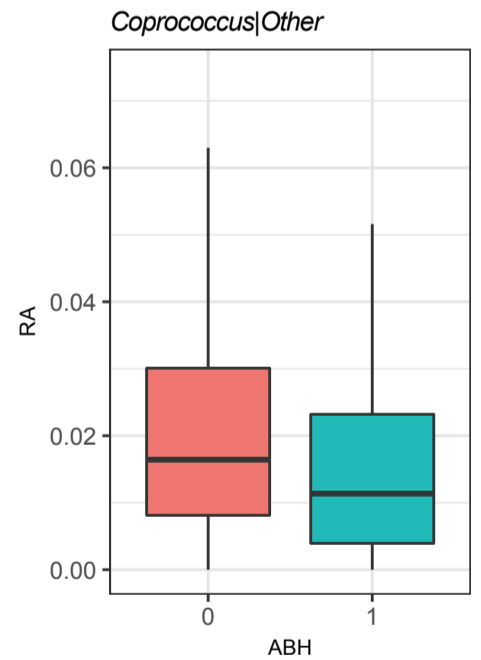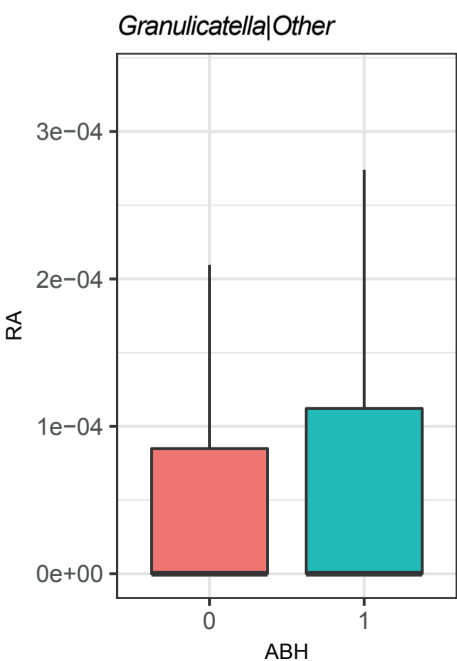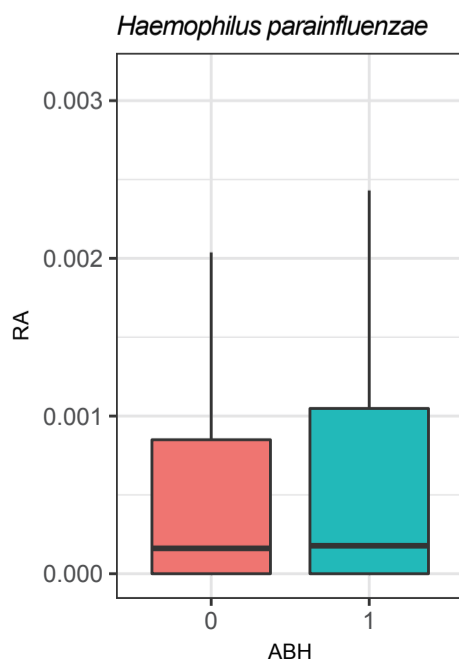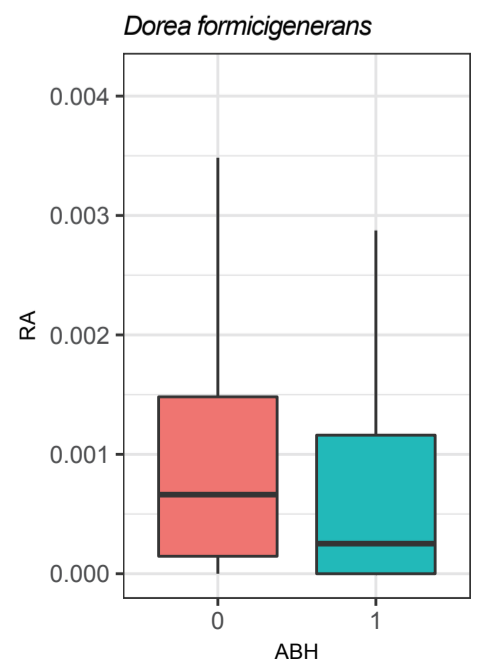

Supplement: Supplementary file 7 — Figure S6. Relative abundances of ABH-associated species present in the fecal samples from AGP subjects without (indicated as 0) or with (indicated as 1) recent antibiotic use (ABH). The species were detected by the proposed two-stage framework OMiAT-HBH and OMiAT-SST. The groups with/without recent antibiotic exposure had 761 and 373 subjects, respectively. (PDF 930 kb) [file 40168_2018_517_MOESM7_ESM.pdf]

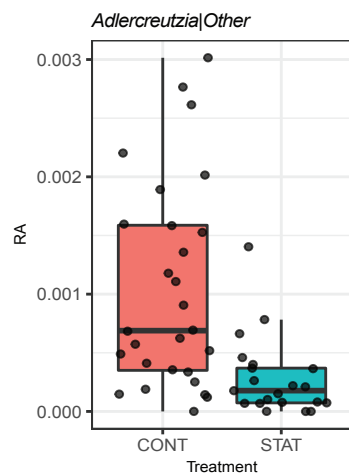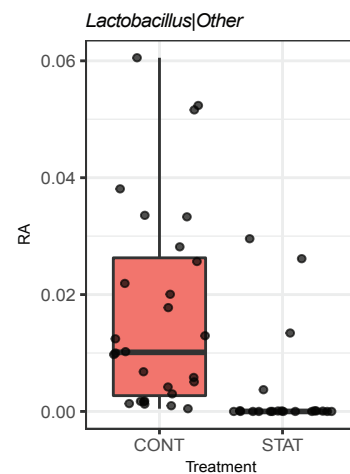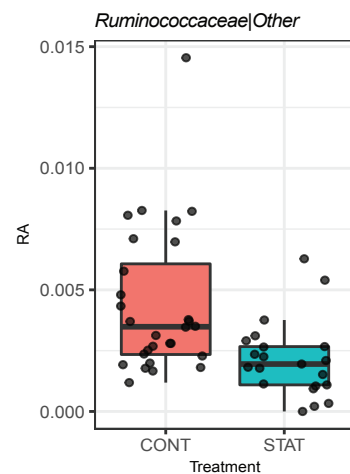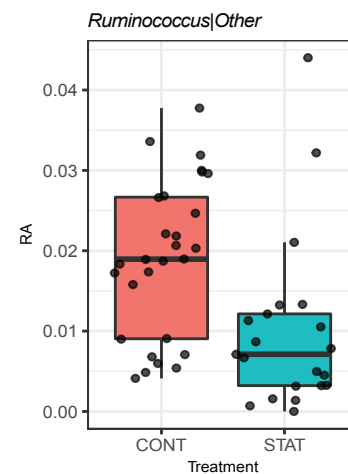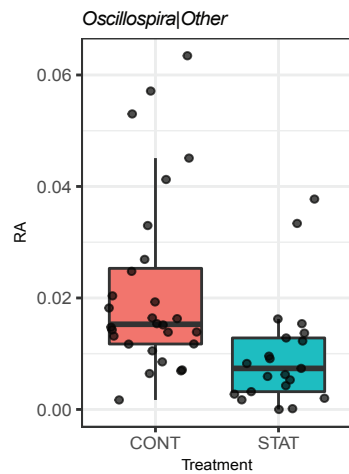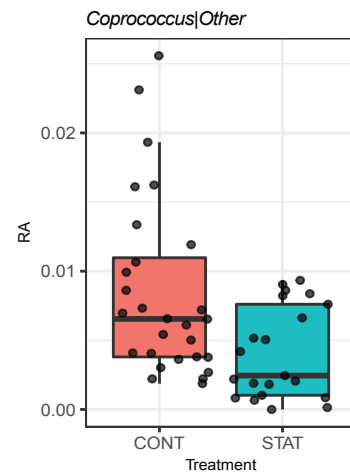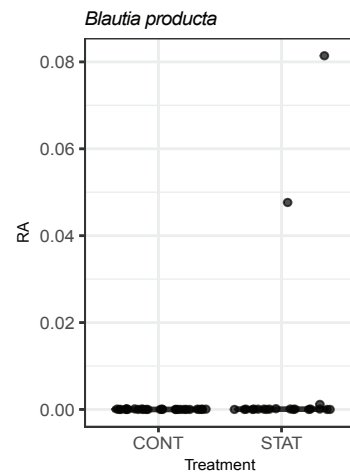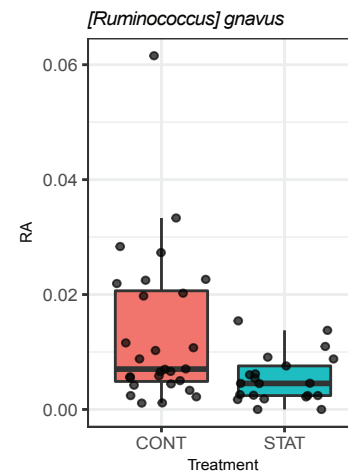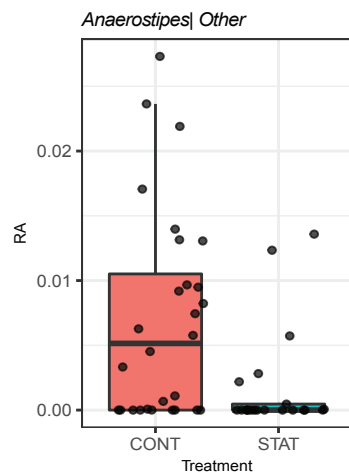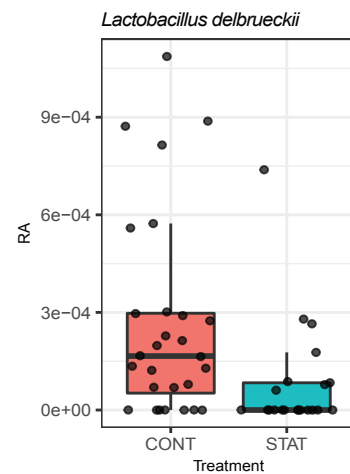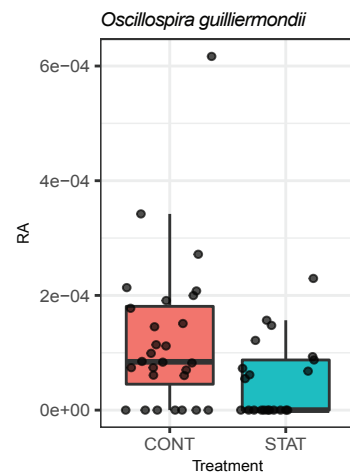

Supplement: Supplementary file 12 — Figure S8. Comparison of the relative abundances (RA) of the significant species between control (CONT) and STAT (STAT) groups. The species were detected by massMap: OMiAT-HBH and OMiAT-SST (FDR = 0.05). (PDF 1883 kb) [file 40168_2018_517_MOESM12_ESM.pdf]
