# Supplementary material for: A two-stage microbial association mapping framework with advanced FDR control
Source: Microbiome. 2018 Jul 25;6:131. doi: 10.1186/s40168-018-0517-1 (PMC6060480; doi:10.1186/s40168-018-0517-1)
Supplement: Supplementary file 6 — Table S1. Candidate taxonomic groups at the family rank associated with a history of recent antibiotic use (ABH) in the AGP data analysis. Groups were detected either by OMiAT or by the aggregate method, respectively. FDR = 0.05. (PDF 91 kb) [file 40168_2018_517_MOESM6_ESM.pdf]

| Family | Size[1] | OMiAT | | Aggregate Method | |
|---|---|---|---|---|---|
| | | Raw p-value | Adjusted p-value | Raw p-value | Adjusted p-value |
| *Ruminococcaceae* | 5 | 5.0E-06 | 1.0E-02 | 2.1E-04 | |
| *Erysipelotrichaceae* | 4 | 1.0E-04 | 3.5E-01 | 3.1E-03 | |
| *Micrococcaceae* | 2 | 4.0E-04 | 1.0E-04 | 5.5E-03 | 5.9E-03 |
| *Lachnospiraceae* | 12 | 1.0E-03 | 3.4E-01 | 9.8E-03 | |
| *Christensenellaceae* | 1 | 1.2E-03 | 1.2E-03 | 1.0E-02 | 2.5E-02 |
| *Porphyromonadaceae* | 3 | 1.6E-03 | 5.1E-01 | 1.1E-02 | |
| *Streptococcaceae* | 1 | 3.5E-03 | 3.5E-03 | 1.4E-02 | 3.5E-02 |
| *Actinomycetaceae* | 1 | 3.4E-03 | 3.4E-03 | 1.4E-02 | 3.5E-02 |
| *[Odoribacteraceae]* | 2 | 3.2E-03 | 4.4E-02 | 1.4E-02 | |
| *Enterobacteriaceae* | 4 | 3.2E-03 | 3.7E-01 | 1.4E-02 | |
| *Carnobacteriaceae* | 1 | 8.1E-03 | 8.1E-03 | 2.8E-02 | |
| *Pasteurellaceae* | 1 | 8.1E-03 | 8.1E-03 | 2.8E-02 | |

[1]The number of species within the corresponding taxonomic group.
