# Supplementary material for: A two-stage microbial association mapping framework with advanced FDR control
Source: Microbiome. 2018 Jul 25;6:131. doi: 10.1186/s40168-018-0517-1 (PMC6060480; doi:10.1186/s40168-018-0517-1)
Supplement: Supplementary file 8 — Table S2. The family groups that were associated with BMI detected by OMiAT in AGP data (FDR = 0.10). The aggregated method did not identify any significant groups at the family rank. (PDF 143 kb) [file 40168_2018_517_MOESM8_ESM.pdf]

Family	Size[1]	OMiAT	
		Raw p-value	Adjusted p-value
Bifidobacteriaceae	2	4.9E-05	2.0E-03
Clostridiaceae	2	1.3E-03	2.7E-02
Erysipelotrichaceae	4	2.9E-03	4.0E-02
Prevotellaceae	4	5.0E-03	5.2E-02
[Barnesiellaceae]	1	1.1E-02	8.7E-02

[1]The number of species within the corresponding taxonomic group.
