# Supplementary material for: A two-stage microbial association mapping framework with advanced FDR control
Source: Microbiome. 2018 Jul 25;6:131. doi: 10.1186/s40168-018-0517-1 (PMC6060480; doi:10.1186/s40168-018-0517-1)
Supplement: Supplementary file 9 — Figure S7. The relative abundances of BMI-associated species for AGP subjects grouped by their BMI quantile. The species shown had been detected by the proposed two-stage framework OMiAT-HBH and OMiAT-SST (FDR = 0.10). (PDF 309 kb) [file 40168_2018_517_MOESM9_ESM.pdf]

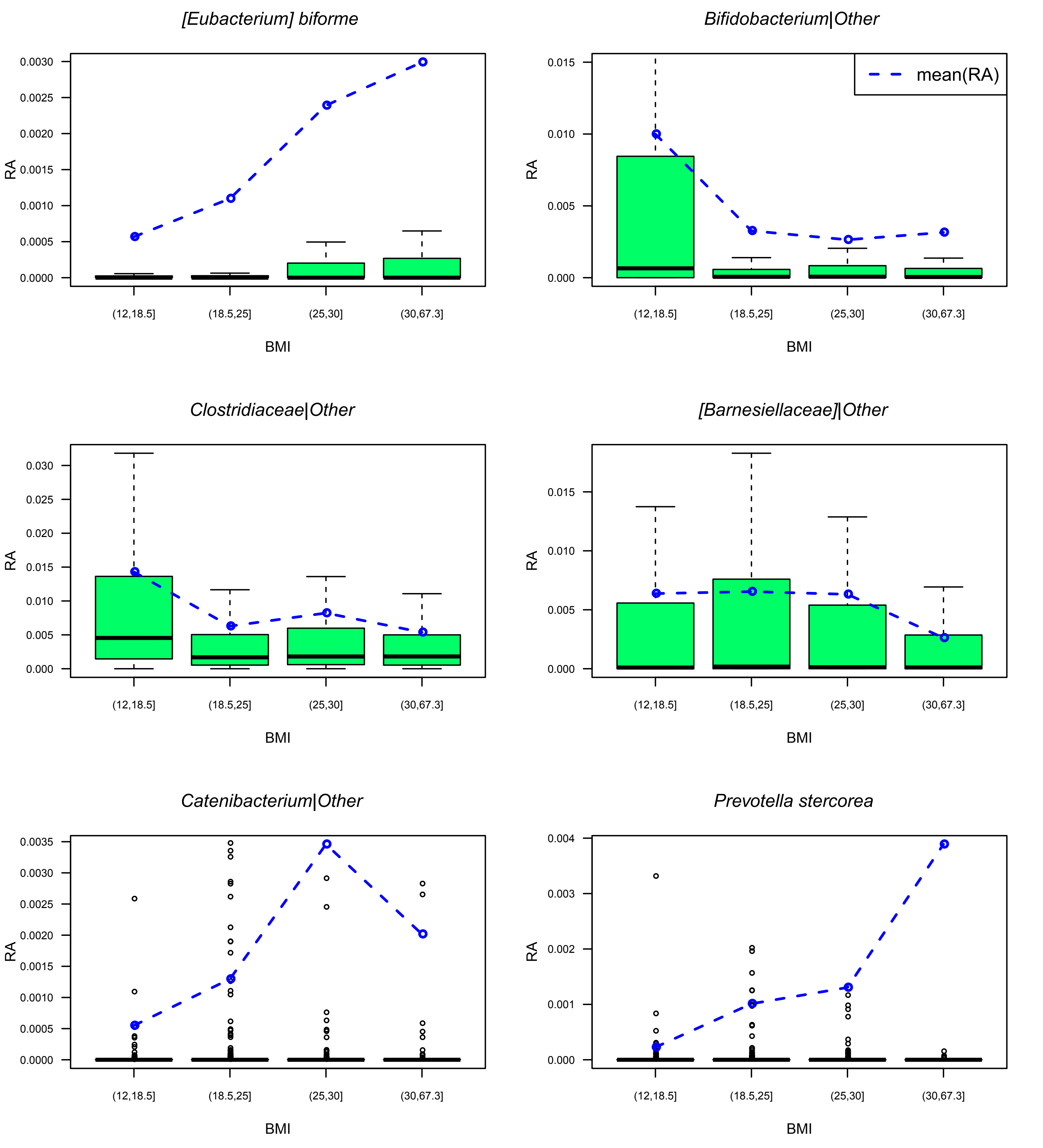

[Eubacterium] biforme
Bifidobacterium|Other
mean(RA)
Clostridiaceae|Other
[Barnesiellaceae]|Other
Catenibacterium|Other
Prevotella stercorea
RA
BMI
(12,18.5]
(18.5,25]
(25,30]
(30,67.3]
