# Supplementary material for: A two-stage microbial association mapping framework with advanced FDR control
Source: Microbiome. 2018 Jul 25;6:131. doi: 10.1186/s40168-018-0517-1 (PMC6060480; doi:10.1186/s40168-018-0517-1)
Supplement: Supplementary file 10 — Table S3. The unadjusted and FDR-adjusted p values for the detected BMI-associated species using the AGP data (FDR = 0.05). Adjusted p values ≥0.05 are left blank. There were no associated species identified by the aggregated method. (PDF 155 kb) [file 40168_2018_517_MOESM10_ESM.pdf]

OTU ID	Species	Raw p-value	BH	OMiAT-HBH	OMiAT-SST	AGG-HBH	AGG-SST
297635	*[Eubacterium] biforme*	1.90E-04	1.70E-02	1.50E-03	7.60E-04		
824876	*Bifidobacterium| Other*	2.70E-03		1.10E-02	5.30E-03		
4319938	*Clostridiaceae| Other*	1.00E-02		2.70E-02	2.00E-02		
**Number of detected BMI-associated species**			**1**	**3**	**3**	**0**	**0**
