# Supplementary material for: A two-stage microbial association mapping framework with advanced FDR control
Source: Microbiome. 2018 Jul 25;6:131. doi: 10.1186/s40168-018-0517-1 (PMC6060480; doi:10.1186/s40168-018-0517-1)
Supplement: Supplementary file 11 — Table S4. Candidate groups (at the family rank) associated with STAT exposure, detected by OMiAT or by the aggregated method, respectively, using data from a murine experiment [28] (FDR = 0.05). (PDF 85 kb) [file 40168_2018_517_MOESM11_ESM.pdf]

Family	Size[1]	OMiAT		Aggregate Method	
		Raw p-value	Adjusted p-value	Raw p-value	Adjusted p-value
Lactobacillaceae	4	5.00E-06	9.00E-05	2.36E-02	
Ruminococcaceae	6	4.00E-04	2.30E-03	2.00E-04	2.50E-03
Coriobacteriaceae	1	3.00E-04	2.30E-03	3.00E-04	2.50E-03
Lachnospiraceae	7	3.40E-03	1.51E-02	9.50E-03	

[1]The number of species within the corresponding taxonomic group.
